# The Role of Organosulfur Compounds as Nrf2 Activators and Their Antioxidant Effects

**DOI:** 10.3390/antiox11071255

**Published:** 2022-06-26

**Authors:** Melford Chuka Egbujor, Maria Petrosino, Karim Zuhra, Luciano Saso

**Affiliations:** 1 Department of Chemical Sciences, Rhema University Nigeria, Aba 453115, Abia State, Nigeria; 2 Department of Pharmacology, Faculty of Science and Medicine, University of Fribourg, 1700 Fribourg, Switzerland; maria.petrosino@unifr.ch (M.P.); karim.zuhra@unifr.ch (K.Z.); 3 Department of Physiology and Pharmacology “Vittorio Erspamer”, Sapienza University of Rome, 00185 Rome, Italy; luciano.saso@uniroma1.it

**Keywords:** organosulfur compounds, Nrf2, antioxidant, anti-inflammation

## Abstract

Nuclear factor erythroid 2-related factor 2 (Nrf2) signaling has become a key pathway for cellular regulation against oxidative stress and inflammation, and therefore an attractive therapeutic target. Several organosulfur compounds are reportedly activators of the Nrf2 pathway. Organosulfur compounds constitute an important class of therapeutic agents in medicinal chemistry due to their ability to participate in biosynthesis, metabolism, cellular functions, and protection of cells from oxidative damage. Sulfur has distinctive chemical properties such as a large number of oxidation states and versatility of reactions that promote fundamental biological reactions and redox biochemistry. The presence of sulfur is responsible for the peculiar features of organosulfur compounds which have been utilized against oxidative stress-mediated diseases. Nrf2 activation being a key therapeutic strategy for oxidative stress is closely tied to sulfur-based chemistry since the ability of compounds to react with sulfhydryl (-SH) groups is a common property of Nrf2 inducers. Although some individual organosulfur compounds have been reported as Nrf2 activators, there are no papers with a collective analysis of these Nrf2-activating organosulfur compounds which may help to broaden the knowledge of their therapeutic potentials and motivate further research. In line with this fact, for the first time, this review article provides collective and comprehensive information on Nrf2-activating organosulfur compounds and their therapeutic effects against oxidative stress, thereby enriching the chemical and pharmacological diversity of Nrf2 activators.

## 1. Introduction

The biological role of small sulfhydryl (-SH) molecules has been widely recognized due to their ability to maintain cellular redox homeostasis [1]. Among them, thioredoxin (Trx), glutathione (GSH) and glutaredoxin are endogenous sulfur-containing molecules that have been described to play a key role in maintaining the redox balance of biological systems via multiple mechanisms, including activation of the Nrf2 pathway [2]. The participation of organosulfur compounds in redox biochemistry, combined with low toxicological profiles and therapeutic effects, make them promising molecules for pharmacological applications [3]. In the last few decades, the relevance of the employment of Nrf2 activators as a potential approach for the treatment of various chronic diseases has been highlighted [4]. The mechanism of action of many Nrf2 activating compounds relies on their ability to interact with Keap1 (Kelch-like ECH associated protein 1), which is a negative modulator of Nrf2. Once activated, Nrf2 mediates the transcription of a wide range of genes involved in maintaining the cellular redox balance, reducing inflammation and promoting mitochondrial respiration and biogenesis [4]. Therefore, drugs able to interfere with Nrf2–Keap1 interaction are of great interest for the treatment of such diseases. Despite the advancement in research for the discovery of Nrf2 activators, only a few approved Nrf2 activators are available [5,6,7,8], such as sulforaphane, oltipraz, ergothioneine, and L-carbocysteine [2,9,10,11,12,13,14,15].

The present review explores recent studies on the activities of organosulfur compounds as activators of the Nrf2 signaling pathway and their potency in preventing or attenuating oxidative stress-mediated diseases.

### 1.1. Role of Nrf2 in Human (Patho)Physiology

**Figure 1:**
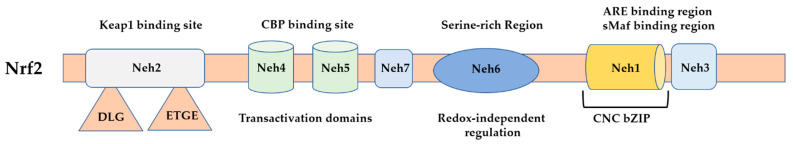
**Domain organization of the transcription factor Nrf2.** Nrf2 is characterized by seven highly conserved regions, Neh1 to Neh7. Neh1, Neh3, and Neh6 are located at the C-terminus of Nrf2, while Neh2, Neh4, and Neh5 are located in the N-terminal region. Neh1 contains a CNC-bZIP domain responsible for dimerization with small musculoaponeurotic fibrosarcoma (Maf) proteins and required for binding to ARE sequences in DNA. Neh2 domain controls the interaction with the cytoplasmic protein Keap1 via DLG and ETGE motifs. The Neh3 domain is required for the activation of transcription, along with the Neh4 and Neh5 domains that enhance Nrf2-mediated reporter gene transcription in an ATP-dependent manner. Neh4 and Neh5 domains are known as transactivation domains and are involved in the binding to the kinase-inducible domain interacting (KIX) and cysteine/histidine-rich domain 3 (CH3) domains of CBP (CREB (cAMP Responsive Element Binding protein)-binding protein). The Neh6 domain contains a serine-rich conserved region which is involved in degradation of Nrf2. This domain contains two binding sites (DSGIS and DSAPGS motifs) for the β-transducin repeat-containing protein (β-TrCP). Finally, the Neh7 domain interacts with the retinoic X receptor α, an Nrf2 repressor, and represses Nrf2 target gene transcription.

Nrf2 is a critical transcription factor discovered in 1994 as a member of the human cap ’n’ collar basic-region leucine zipper (bZip) family. It contains: (i) a highly conserved C-terminal basic zipper domain, which is required for protein–protein interactions as well as for binding to DNA; (ii) a small N-terminal negative regulatory domain, named Neh2, responsible for the interaction of Nrf2 with the Keap1 protein; and (iii) a large transcriptional activation domain which promotes the recruitment of transcriptional co-activators to an enhancer sequence in the promoter region of genes known as antioxidant response element (ARE) (Figure 1) [16,17,18,19,20].

Follow-up studies in Nrf2-KO mice established that Nrf2 takes part in the regulation of about 250 genes containing ARE. These genes encode enzymes involved in the antioxidant response, thus playing a crucial role in attenuating oxidative stress, inflammation, and apoptosis [21,22,23,24,25,26,27]. Nrf2 is now widely accepted as the master regulator of stress response [28,29] and the cellular defence system and overall it has a key role in maintaining cellular homeostasis [30,31,32]. For instance, Nrf2 has been shown to support the Trx/GSH system, a major defence against ROS generation and redox imbalance in cell and tissues. Indeed, it has been established that Nrf2 induces increased expression levels of thioredoxin, thioredoxin reductase, and peroxiredoxin, involved in the reduction of oxidized cysteines of target proteins and redox signal transduction [33]. Moreover, Nrf2 stimulates GSH *de novo* biosynthesis and GSH recycling by increasing the expression levels of glutamate–cysteine ligase (GCL) and glutathione reductase [33,34,35]. Activation of the Nrf2/ARE pathway has been shown to increase the generation levels of NADPH, an important source of reducing equivalents involved in many redox reactions [1]. Nrf2 has been reported to promote the expression of enzymes involved in quinone redox cycling such as NADPH quinone oxidoreductase 1 (NQOI) [36,37,38]. The activation of the Nrf2/ARE signaling pathway correlates with increased expression levels of heme oxygenase-1 (HO-1), which contributes to the elimination of ROS by generating the antioxidant biliverdin and catalysing the degradation of “free” heme, which has prooxidant properties [39]. Nrf2 attenuates the inflammatory response by inhibiting the transcriptional activity of NF-κB and by suppressing pro-inflammatory cytokines [40,41]. Moreover, Nrf2 regulates the expression levels of cystathionine β-synthase (CBS) and cystathionine γ-lyase (CSE), the major enzymatic source of hydrogen sulfide (H_2_S), as well as sulfide:quinone oxidoreductase (SQR), involved in its catabolism [42].

Nrf2 has also been shown to have cytoprotective effects by playing pivotal roles in preventing xenobiotics-induced toxicity and carcinogen-related tumorigenesis. These protective effects are mainly attributed to the activation of genes that encode for enzymes acting in detoxification and/or in the transport of various toxic xenobiotics, thus leading to their excretion from the body [43,44,45,46]. Indeed, a cross-talk between Nrf2/ARE dependent signaling and Ahr-XRE signaling has been recently demonstrated [47,48]. The Nrf2 promoter has three xenobiotic response element (XRE)-like element (XREL) regions, XREL1, XREL2, and XREL3, to which the aryl hydrocarbon receptor–xenobiotic response element (AhR) binds and causes transactivation of the AhR–XRE signaling pathway. AhR is a transcription factor that regulates gene expression of crucial xenobiotic-metabolizing enzymes in response to various chemicals; when activated it induces the transcription of its dependent genes including various cytochrome P450s (CYPs) [49] and drives their transcription [50] thus leading to a protective response against a wide range of xenobiotics.

The broad-spectrum effect of the activation of the Nrf2/ARE pathway on redox metabolism and its “multi-target” cytoprotective effect can be exploited for the treatment of chronic multifactorial disorders characterized by the lack of a unique aetiology. As extensively reviewed by Cuadrado et al. [4], pharmacological activators of the Nrf2 pathway may prove beneficial for treatment of respiratory, metabolic, cardiovascular, and neurodegenerative diseases. However, in cancer the role of Nrf2 is controversial. On the one hand, it has been shown that Nrf2 activation induces a transcriptional response which is responsible for maintaining low ROS levels and repairing oxidative damage, thus preventing tumorigenesis. On the other hand, several lines of evidence suggest that constitutive increased levels of Nrf2 promotes cancer progression, metastasis, and chemoresistance [51]. To date, the role of Nrf2 in cancer biology has not been clarified, as Nrf2 can act both as a tumour suppressor and an oncogenic protein. Therefore, from the pharmacological point of view, Nrf2 activators can be employed only in cancer prevention, whereas the development of Nrf2 inhibitors is under evaluation as a strategy for cancer treatment.

### 1.2. Nrf2 Regulation

**Figure 2:**
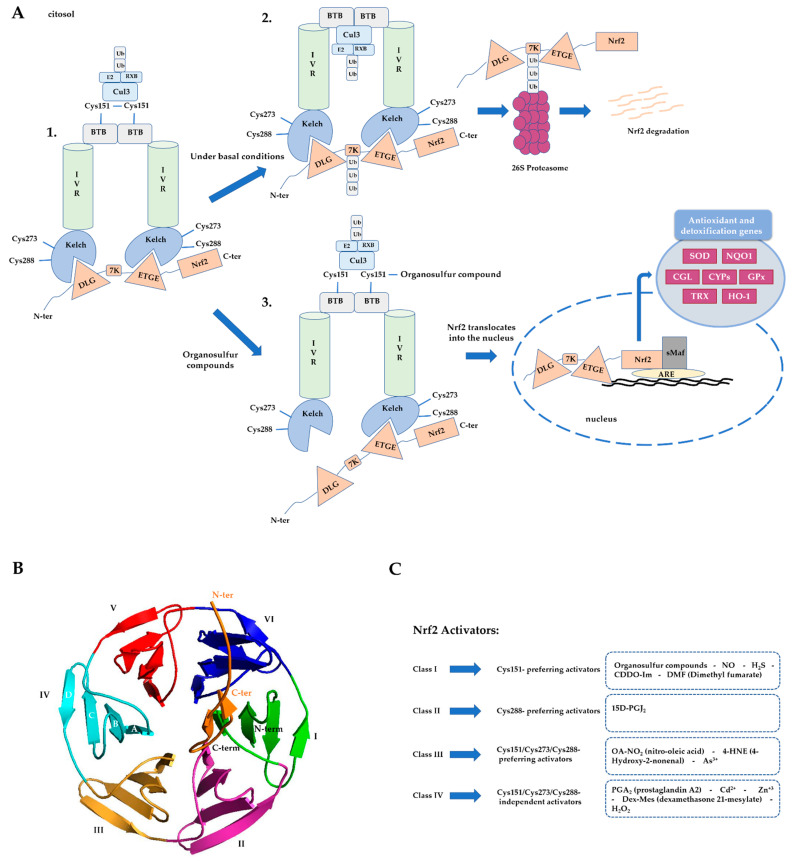
(**A**) **Regulation of Nrf2 activity by Keap1.** (**1.**) Keap1 in dimeric form, complexed with Nrf2. (**2.**) Under basal conditions, Nrf2 interacts with Keap1 homodimer through ETGE and DLG motifs. This interaction allows the ubiquitination of Nrf2 at its Lys-rich (7K) region and the following proteasomal degradation of the protein by the 26S proteasome. (**3.**) The presence of Nrf2 activators (such as organosulfur compounds) promotes the release of Nrf2 thus leading to its nuclear translocation. Particularly, activators react with specific cysteine residues in Keap1 (Cys151, Cys273, or Cys288). In the nucleus, Nrf2 heterodimerizes with small Maf (sMaf) proteins and binds to the antioxidant response element (ARE), activating the expression of a battery of cytoprotective genes. (**B**) **Structure of the Kelch domain of human Keap1 in complex with Nrf2 peptide (pdb #4IFL).** The Kelch domain of Keap1 is characterized by a six-bladed β-propeller structure. Each blade, shown in a different color and numbered I–VI, is composed of four β-strands labeled A–D (as shown in white font on blade IV). The N and C termini are both located in blade I. The N and C termini of Nrf2 peptide are also indicated in figure. (**C**) **Scheme of chemical Nrf2 activators including some organosulfur compounds.** Four different classes of Nrf2 activators and representative chemicals for each class are reported. Class I contains the Cys151-preferring activators, class II the Cys288-preferring activators, class III the Cys151/Cys273/Cys288-preferring activators, and class IV the Cys151/Cys273/Cys288-independent activators.

Nrf2 is constitutively expressed in cells and tissues under homeostatic conditions, thus providing basal expression levels of its target genes. It displays a half-life of less than 10 min due to permanent degradation by the ubiquitin proteasome system. However, under stress conditions, Nrf2 degradation slows down, thus leading to its accumulation in the nucleus [52] (Figure 2A). This adaptive system ensures higher Nrf2 levels under stressing conditions, providing enhanced expression levels of ARE-dependent antioxidant genes and attenuating oxidative stress and inflammation. Nrf2 turnover is regulated by Keap1 (Figure 2A). Keap1 is a cytosolic protein consisting of three domains, the BTB domain (Broad complex, tramtrack and bric-à-brac domain), an intervening region (IVR), and a C-terminal Kelch domain. The Kelch domain of Keap1 folds up into a six-bladed β-propeller structure [53], which is a disc-like structure assembled by circularly arranged structural modules (named blades) with a small channel that runs through the center. Each blade is a twisted β-sheet composed of four antiparallel β-strands (A–D): strand A is positioned in line with the central channel and runs almost parallel with the six-fold axis of the propeller, while strands B–D progressively acquire more distance from the central axis and exhibit more twist until almost complete perpendicularity to the six-fold axis is reached with strand D that lies outside the edge of the protein (Figure 2B) [54,55]. A stable interface between blade I and VI allows the formation of an arrangement that closes the ring of the blades in the propeller, known as C-terminal strand mechanism of closure. Keap1 dimerization is required for Keap1–Nrf2 association, thus dimeric Keap1 combines with Nrf2 with a 2:1 stoichiometry [56,57] to form a Nrf2-Keap1 complex which, under normal conditions, undergoes ubiquitination and proteasomal degradation in the cytoplasm. This association occurs through the recognition of the Neh2-domain in the Nrf2 protein. The Neh2 domain of Nrf2 is a small N-terminal negative regulatory domain of the transcription factor which mediates binding of Nrf2 to the cytoplasmic Keap1 protein. Indeed, Neh2 is stretched between two Kelch domains in the Keap1 homodimer and binds Kelch via two different binding motifs, one with low affinity (the DLG domain) and one with high affinity (the ETGE domain) (Figure 1 and Figure 2A), which is composed of an evolutionarily conserved amino acid sequence motif [57,58]. The DLG motif is characterized by three helices that weakly bind to the double glycine domain of Keap1. By contrast, the ETGE motif, characterized by a single β-hairpin structure, is able to bind the double glycine domain of Keap1 in a key-and-lock manner [58,59,60]. The binding of these two motifs of Nrf2 to the Keap1 homodimer allows the lysine residues in Neh2-domain to be ubiquitinylated by Cullin3 (Cul3) ligase and thus leading Nrf2 labeled for proteasomal degradation. Keap1 activity is regulated by redox-sensitive cysteine residues exposed to the solvent, which, under oxidative stress, are modified by electrophiles, thus leading to the release of Nrf2 [61,62] (Figure 2A). The most common pharmacological approach to activate Nrf2 is to prevent its Keap1-dependent degradation. Indeed, many Nrf2 activators are actually Keap1 inhibitors by targeting the redox-sensitive cysteines [63,64]. Among them, Cys151 plays a central role in the regulation of the Keap1 pathway by (i) mediating the dimerization of Keap1 to its biologically active homodimer and (ii) stabilizing the Keap1–Cul3 complex. Consistently, compounds reactive towards Cys151 of Keap1 (also referred as Nrf2 activators of Class I, Figure 2C) elicit a powerful Nrf2-activator effect by preventing Keap1 dimerization and promoting detachment of Cul3. In turn, Cys151-mediated regulation of Keap1 has been demonstrated to have a pivotal role in the process leading to Nrf2 ubiquitination and eventually to its degradation. Endogenous signaling molecules such as nitric oxide (NO) and hydrogen sulfide (H_2_S) have been shown to react with Cys151, hence mediating the activation of the Nrf2/ARE pathway [65]. Mechanistic details on the control of Nrf2 by H_2_S and possible effects of sulfane sulfur-containing species (per-/polysulfides) will be discussed. Moreover, although it is commonly accepted that organosulfur compounds lower ROS levels through activation of the Nrf2/ARE pathway, some lines of evidence suggested that this mechanism may be mediated by ROS themselves. For instance, homocysteine, a classical organosulfur compound, exerts controversial effects on the antioxidant defense system. As extensively reviewed in [66], on the one hand homocysteine mediates antioxidant effects through the activation of the Nrf2/ARE pathway (as further discussed in Section 3.2.3), on the other hand elevated homocysteine levels are often associated with ROS-mediated cytotoxicity. This discrepancy could be related to the multiple metabolic pathways through which homocysteine influences cell functions in different conditions and tissues. To add a further grade of complexity, it is also conceivable that homocysteine-derived ROS are able, to a certain extent, to mediate the activation of the antioxidant response. Moreover, it has been shown that garlic-derived organosulfur compounds, such as diallyl sulfide, rapidly increase (after 15–30 min from the treatment) the generation of H_2_O_2_ [67,68], a known activator of Nrf2. This is in apparent contrast with the noxious effect exerted by ROS, but reconciled by the finding that pre-conditioning cells with low levels of H_2_O_2_ confers resistance to oxidative stress [69]. From the mechanistic point of view, the H_2_O_2_-mediated activation of the Nrf2/ARE pathway seems to be Cys151, Cys273, or Cys288 independent (Nrf2 activators of Class IV, Figure 2C). Accordingly, in mouse embryonic fibroblasts (MEFs) harboring triple cysteine sensor mutation on Keap1, in which the three cysteines Cys151, Cys273 and Cys288 were substituted with serine, tryptophan, and glutamic acid, respectively, activation of the Nrf2 pathway in response to Class IV activators (but not Class I–III) was observed [62]. This may suggest that other cysteines than Cys151, Cys273, and Cys288 are involved in the H_2_O_2_-mediated activation of Nrf2. As previously reviewed by Suzuki et al. [70], Cys226, Cys434, and Cys613 may play a role in H_2_O_2_ sensing, although this needs to be further validated. 

Of pharmacological relevance, dimethyl fumarate (DMF), an approved drug for treatment of multiple sclerosis and psoriasis, has been shown to induce Keap1 inactivation through electrophilic modification of Cys151 [71]. Similarly, other organosulfur compounds such as sulforaphane (SFN) and oltipraz or the synthetic terpenoid 1-[2-cyano-3,12-dioxooleana-1,9(11)-dien-28-oyl] imidazole (CDDO-Im) induce a chemical modification of Cys151, thus resulting in the detachment of Cul3 ligase and in the stabilization of Nrf2 (Figure 2A,C) [62,63,72,73]. Furthermore, modification of other redox-active cysteine residues in Keap1, such as Cys288, induces conformational changes leading to the dissociation of Keap1–DLG interaction [63,74]. This mechanism may give reason of the functional consequences of the modification of Cys288 induced by 15d-PGJ2 (15-deoxy-Δ12,14-prostaglandin J2), which has been reported to induce the break of the dimeric structure of Keap1 and the release of Nrf2 (Figure 2C) [62].

It is worthy to note that antioxidant response element (ARE) is the key player in the Keap1/Nrf2/ARE signaling system and essential to the Nrf2-mediated transcription of genes involved in the defense mechanisms of organisms. ARE can be described as a *cis*-acting element or enhancer sequence normally located in the promoter region of series of genes encoding detoxification enzymes. It mediates the transcriptional activation of genes, encoding proteins that control redox homeostasis and prevent oxidative damage [75]. Several transcriptional factors, especially Nrf2, depend on AREs for the regulation of cytoprotective genes [76]. The activation of the Nrf2/ARE pathway has been found to protect endothelial cells from oxidative damage [77]. Under oxidative stress conditions, Nrf2 undergoes dissociation from Keap1 and translocation into the nucleus where it forms heterodimer with small musculoaponeurotic fibrosarcoma (Maf) proteins and binds to ARE in order to effect transcriptional activation of antioxidant genes [78].

## 2. Nrf2-Activating Synthetic Organosulfur Compounds

### 2.1. Sulfonyl Group-Containing Compounds

Sulfonyl group-containing compounds, especially sulfones and sulfonamides, have been widely studied because of their significant therapeutic roles for a number of diseases. Their tense chemical structure and functionality enable them to form hydrogen bonding interaction with active site residues of biochemical targets. Their usual incorporation into a core ring structure gives them specific conformations which fit the active sites [79]. Sulfonyl group-containing compounds exhibit therapeutic effects against bacterial infections [80,81,82,83,84,85,86], tumors [87,88], Alzheimer’s disease [89], oxidative stress-related diseases [90,91,92,93], and diabetes [79]. Moreover, sulfonyl-containing compounds have been found to activate the Nrf2 pathway and inhibit Keap1–Nrf2 protein–protein interaction, thereby alleviating oxidative stress and inflammatory conditions [94].

#### 2.1.1. Sulfone Derivatives

**Scheme 1:**
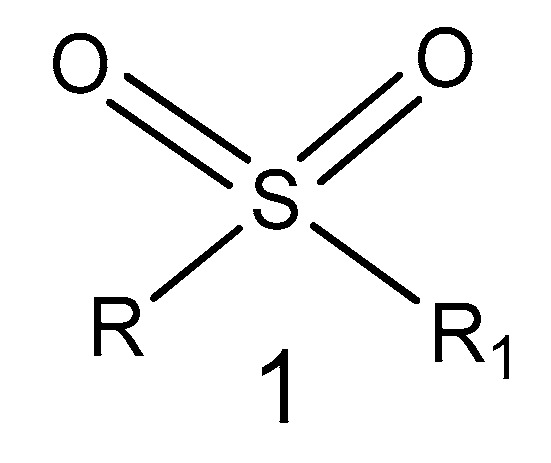
Structure of sulfone.

Sulfone is an organic sulfur compound in which the sulfonyl functional group is bonded to two carbon atoms (Scheme 1). The central sulfur is hexavalent, doubly-bonded to two oxygen atoms and singly-bonded to two carbon atoms [95].

**Scheme 2:**
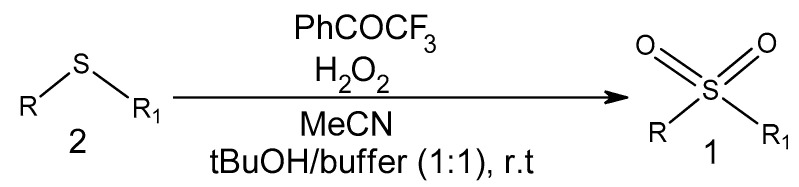
Synthesis of sulfones.

Sulfones (1) are synthesized by organocatalytic oxidation of sulfides (Scheme 2), which remains a common method [95,96].

**Scheme 3:**
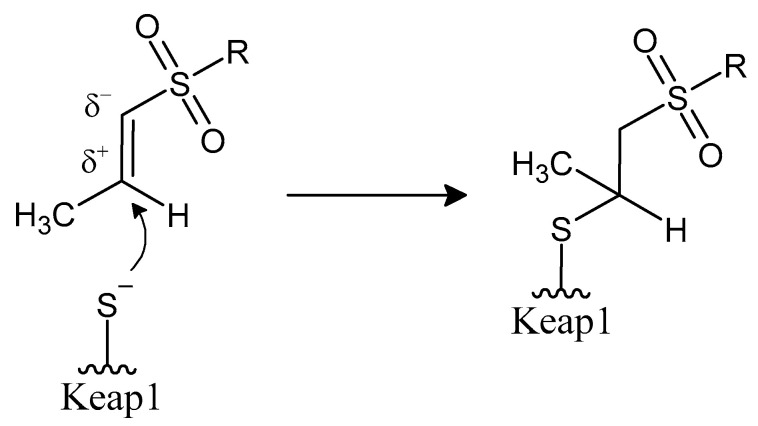
Binding of Keap1 to Vinyl Sulfones.

With the aim of implementing the specificity and lowering off-targets, within the category of sulfones there have been designed molecules sufficiently electrophilic to react with redox sensitive cysteine residues of target proteins, but still unreactive towards low-molecular weight thiols such as glutathione (GSH) (in which case this would lead to depletion of GSH). This is the case, for instance, with electrophilic double-bound bearing vinyl sulfone scaffold: when the substituent R is a vinyl group, the polarized double bond C=C is highly reactive towards protein nucleophilic sulfhydryls [97] (Scheme 3). Vinyl sulfones have been extensively studied as a novel class of neuroprotective agents for Parkinson’s disease therapy due to their significant Nrf2 activation [98,99,100]. The tuning of the electrophilicity and steric hindrance of vinyl sulfones can be harnessed for the development of new potent Nrf2 activators for neuroprotection [32]. From the molecular point of view, the target of this class of compounds has been shown to be Keap1. The direct binding to one of the solvent exposed cysteines of Keap1 prevents complexation with Nrf2 and its consequent degradation [97] (Scheme 3). This has been shown, for instance, with (E)-1-chloro-2-(2-((2-methoxyphenyl)sulfonyl)vinyl)benzene, here referred to as compound **4** (Table 1).

Vinyl sulfones exhibit anti-inflammatory activities [101] and act as modulators of Nrf2 activity [98]. As shown in BV2-microglia cells activated with LPS, Lee et al. [98] reported that vinyl sulfone derivative (**4**) (Table 1) induces Nrf2 transcriptional activity, increases nuclear Nrf2 levels, up-regulates the expression of the Nrf2-dependent antioxidant enzyme genes and down-regulates the production of NO. Particularly, in 1-methyl-4-phenyl-1,2,3,6-tetrahydropyridine (MPTP) mouse model of Parkinson’s disease (PD), oral administration of compound **4** decreased the number of activated microglia in the substantia nigra. This relies on the activation of the Nrf2 pathway, thereby exploiting antioxidant and anti-inflammatory properties, consistent with reduced neuroinflammation [98]. In a similar study, Woo et al. [99] reported that compound **4** activates Nrf2 and induces the expression of the Nrf2–dependent antioxidant enzymes in DAergic neuronal cells at both mRNA and protein levels. Compound **4** exerts a protective effect against cytotoxic damage of DAergic neurons and attenuates Parkinson’s disease-related motor deficits in MPTP-induced mice [99]. Vinyl sulfone is reportedly more potent than chalcone and vinyl sulfoxide in up-regulating the expression of the Nrf2–dependent HO-1 gene [99]. Carlstrom et al. [100] reported that compound **4**, while activating the Nrf2 pathway with a similar potency to DMF, displayed lower off-target effects on NF-ĸB and Hypoxia-inducible factor 1 (HIF1). Choi et al. [102] reported that compound **4** activates Nrf2 and subsequently induces the Nrf2-dependent antioxidant enzyme expression in a dose-dependent manner. In the MPTP-induced Parkinson’s disease model, compound **4** was found to exhibit significant efficacy but displayed poor drug-like properties, such as low solubility, metabolic stability, and permeability through the blood–brain barrier (BBB) [91]. In a follow-up study, Choi and co-workers [102] implemented the Nrf2 activation potency and the ADME/tox profile of compound **4** by incorporating pyridine and morpholine moieties to afford compound **5**. Reportedly, **5** (EC_50_ = 346 nM) is a more potent Nrf2 activator than compound **4** (EC_50_ = 530 nM). Moreover, **5** displayed excellent drug-like properties, showing low toxicity, high microsomal stability, low inhibitory effect on CYP, and good BBB permeability [102]. Compound **5** has been shown also to improve motility in acute MPTP-induced Parkinson’s disease-infected mice, alleviate loss of DAergic neurons, and attenuate microglial activation [102].

Song et al. [32] reported that vinyl sulfone derivatives (compounds **6** and **7**) (Table 1) activate the cellular response system at translational and transcriptional levels. As shown in PC12 cells, compounds **6** and **7** exert cytoprotective effects against oxidative stress via Nrf2 activation and consequent nuclear translocation of Nrf2. The Nrf2-mediated up-regulation of antioxidant species such as HO-1, TrxR, GSH, and NQO1 induced by compounds **6** and **7** protects PC12 cells from H_2_O_2_-mediated damage [32].

Choi and co-workers [102] reported that the introduction of *o*-pyridine as a heterocycle into the ring B of compound **4** (Table 1) reduces Nrf2 activation by 17-fold while the addition of Cl- group to the *o*-pyridine improves the Nrf2 activation of compound **4**. It was observed that the addition of a Cl- group to the ortho position induces the highest Nrf2 activity while substituting the Cl- group with F- results to a six-fold decrease in Nrf2 activation. It was also reported that the introduction of methoxy groups at the 2-,3-, and 4- positions of ring A exerts a remarkable Nrf2 activation effect on compound **4** in the order 3-OMe > 2-OMe > 4-OMe. However, 2-methoxy derivatives exhibit the highest HO-1 induction activity and suppression of NO production [98,99]. Although the addition of *o*-, *m*- or *p*-pyridine to the ring B enhances the Nrf2 activation of compound **4** in the order *p*-pyridine>*o*-pyridine>*m*-pyridine, the *p*-pyridine derivatives are more cytotoxic than *m*- and *o*-pyridine derivatives of compound **4** [102].

#### 2.1.2. Sulfonamide Derivatives

The importance of finding novel pharmacological approaches in activating the Keap1–Nrf2 pathway is witnessed by the still growing number of studies reporting high-throughput screening campaigns employing orthogonal methods ranging from biophysical and bioinformatic approaches, complemented with x-ray crystallography. For instance, in PubChem Bioassay database are reported 528 compounds obtained from high-throughput screens of hundreds of thousands of compounds. Among the confirmed hits, some sulfonamide compounds showed in vitro potency below 100 nM, thus representing valuable pharmacological tools for inducing the activation of the Nrf2/Keap1 pathway [103].

**Scheme 4:**
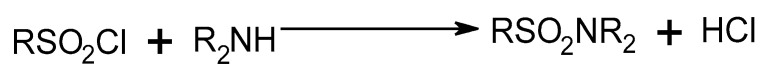
Synthesis of sulfonamides.

Sulfonamides are a group of synthetic medicinal compounds in which a sulfonyl group is bound to an amine group. They have the functional group –S(=O)_2_-NH_2_ and general formula RSO_2_NH_2_ [104]. Sulfonamides can be prepared using several laboratory methods but the reaction of sulfonyl chlorides with amines or ammonia remains the classic synthetic method [105] (Scheme 4).

**Figure 3:**
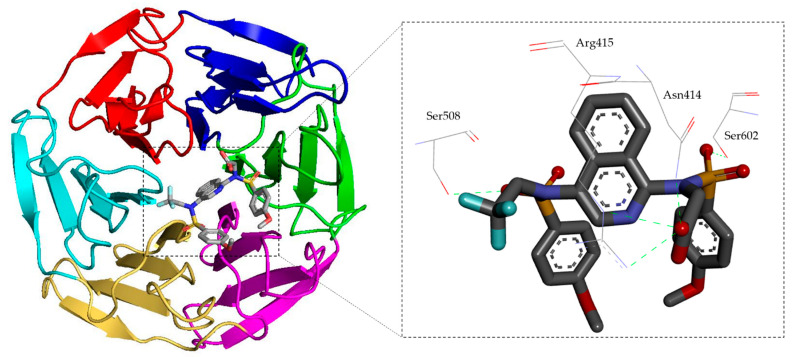
**Crystal structure of compound 14 in complex with human Keap1 Kelch domain (pdb #6UF0).** Overall structure of Keap1 Kelch domain in complex with compound **14** and details of interaction (inset).

Sulfonamides exhibit a broad spectrum of biological effects such as antioxidant [106], anticancer [107] and anti-inflammatory [108] activities. Sulfonamide derivatives have been found to activate Nrf2 and inhibit Keap1-Nrf2 protein-protein interaction thereby antagonizing oxidative stress. This results in the elimination of reactive oxygen species (ROS), alteration of glutathione homeostasis, up-regulation of the expression of antioxidant response element dependent genes and enhancement of antioxidant defence mechanism [1,109,110]. Peng and co-workers [111] reported that caffeic acid sulfonamide derivatives (**8**, **9**, **10**) (Table 1), when used in the treatment of H_2_O_2_-stimulated A549 cells, enhance mRNA expression of Nrf2 and its target genes. Naphthalene sulphonamide derivatives compound are notable Nrf2 activators. Compound **11** activates Nrf2 pathway by inhibiting the Keap1-Nrf2 protein-protein interaction [94]. Compound **12** stimulates Nrf2 protein expression, enhances the anti-oxidative mechanism in macrophage-like RAW264.7 cells and activates Nrf2/ARE pathway in HepG2-ARE-C8 cells [94]. Compound **13** and **14** are potent Keap1-Nrf2 inhibitors which displace the Nrf2 ETGE peptide, enhance the transcription of the Nrf2 targeting genes and activate the Nrf2/ARE signaling pathway [59,94,112,113]. In particular, both inhibit the Keap1-Nrf2 interaction via competing with Helix-2 of DLG for binding to Keap1 [94,113]. As shown in the crystal structure of human Keap1 Kelch domain complexed with compound **14** (Figure 3), this compound binds aminoacidic residues (Ser508, Arg415, Asn414 and Ser602) of the Keap1 Kelch domain, thus seating at the interface between Keap1 and Helix-2 of Nrf2 [59,94]. Naphthalene-based sulfonamides have proven to be promising Nrf2 activators and medicinal chemistry studies have been carried out. Interestingly, it has been shown that compound **13** when substituted with R = OH displays a K_D_ of 3.6 nM, thus being among the most powerful compounds found to inhibit protein-protein interaction between Keap1 and Nrf2 [94,103]. Instead, substitution with R = NH_2_ lowers the binding affinity by about 10-fold.

Lu and co-workers [94] reported that the addition of a hydrophobic benzene ring to naphthalene sulfonamide derivatives (compound **15**) (Table 1) enhances the Nrf2 activating efficiency. The presence of a methoxy group enhances the Keap1–Nrf2 protein–protein interaction (PPI) inhibition and the induction of the Nrf2/ARE pathway, while the absence of methoxy group in sulfonamide substituted phenyl ring decreases the biological effect [94]. *P*-methoxy groups confers higher Nrf2-activation potency than both *m*-methoxy group and *p*-methyl group. The addition of *o*-methyl group increases hydrophobic interactions and hydrophobic groups improve the Keap1–Nrf2 protein–protein interaction (PPI) inhibitory effect of sulfonamide derivatives [94]. Recently, Georgakopoulos et al. [114] reported a non-electrophilic phenyl bis-sulfonamide (**16**) as an effective inhibitor of Keap1–Nrf2 protein–protein interaction having an alternative binding mode. Compound **16** binds and interacts with Keap1 cells and improves the expression of Nrf2-mediated genes such as NQO1, HO-1, and GST. Vinyl sulfonamides, another class of Nrf2 activators, have been reported to act as Keap1–Nrf2 PPI inhibitors and can be utilized as preventive and therapeutic agents for oxidative stress and inflammation (compound **17**). Choi et al. [115] reported that the addition of a methoxy group and chloride to the 2-position of the ring A and 2-position of the ring B increases the Nrf2 activation of vinyl sulfonamides (compound **18**). 

#### 2.1.3. Sulfonate Derivatives

**Scheme 5:**
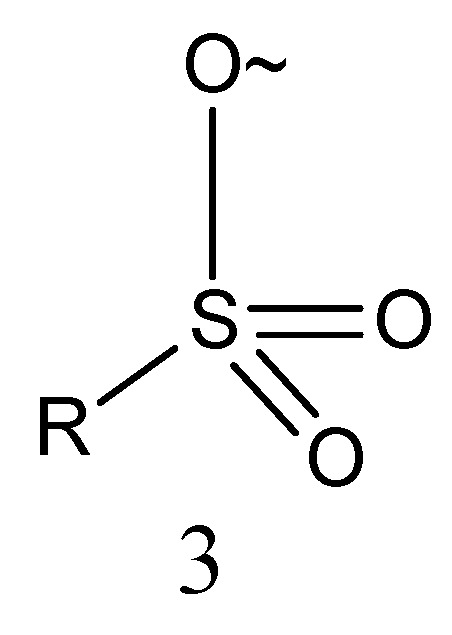
Sulfonate ion.

Sulfonate is a salt of a sulfonic acid with the functional group R-SO_3_^−^ [116] (Scheme 5). Sulfonates are colorless non-oxidizing salts that are generally stable in water [116], they are found in many useful biochemicals and dietary sugars such as sulfoquinovose [117].

**Scheme 6:**
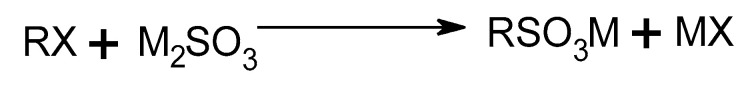
Synthesis of sulfonates.

Sulfonates are synthesized by the reaction of alkyl halides with sulfites usually sodium sulfite (Scheme 6) [117].

Sulfonate derivatives exhibit significant antioxidant [118] and anti-inflammatory effects [119,120]. Vinyl sulfonates (compounds **19**, **20**, and **21**) (Table 1) have been reported as excellent Nrf2 activators with therapeutic potentials for the treatment of Parkinson’s disease [115]. These compounds have been confirmed as potent antioxidant, anti-inflammatory, and neuroprotective agents via both in vivo and in vitro studies [115]. For instance, compound **19** activates the Nrf2/ARE signaling pathway in SH-SY5Y and BV-2 cells and significantly increases the NQO1, GCL, and GCLM protein levels. Compound **19** also attenuates LPS-induced inflammation in BV-2 cells, suppresses the up-regulation of iNOS and COX-2 and decreases the LPS-induced NO production [115]. Moreover, the expression of Nrf2-dependent antioxidant enzymes induced by compound **19** has been shown to prevent motor deficits in MPTP-induced Parkinson’s disease and production of inflammatory mediators. In the same study, the Nrf2-activating efficacy of different classes of organosulfur compounds was compared, with compound **19** (EC_50_ = 76 nM) exhibiting higher Nrf2 activation than vinyl sulfone (EC_50_ = 530 nM) and sulforaphane (EC_50_ = 580 nM) [115]. Structure activity relationship of sulfonates, especially vinyl sulfonates, showed that they exhibit the highest Nrf2 activation when 2-Ome (2-Ome > 3-Ome > 4-Ome) is introduced to ring A and 2-Cl (2-Cl > 3-Cl > 4-Cl) to ring B [115]. An interesting organosulfur compound is sodium tanshinone IIA sulfonate (**22**), a major component of the traditional Chinese herb Danshen (Salvia miltiorrhiza). Due to its beneficial properties, this compound has been used for the treatment of oxidative stress-related diseases. Zhu and co-workers [121], in an in vitro model of silicosis, have shown that the beneficial effects of compound **22** are associated, at least in part, with the activation of the Nrf2 pathway. Particularly, in a coculture of macrophages Raw 264.7 with lung fibroblasts MRC-5, it has been shown that compound **22** inhibited the H_2_O_2_- and silica-induced MRC-5 cell proliferation, thus showing anti-fibrotic properties. Moreover, compound **22** suppressed silica-induced ROS production, decreased the expression of collagen I and collagen III, and increased the thioredoxin and thioredoxin reductase protein expression in MRC-5 cells. Interestingly, all these events correlated with increased nuclear translocation of Nrf2, increased Nrf2 expression levels and up-regulation of the ARE genes [121].

### 2.2. Sulfinyl Group-Containing Compounds

The sulfinyl group refers to the SO group present in several organic compounds. This group is a strong electron-withdrawing substituent with a high configurational stability and biological activity [122,123]. The sulfinyl group is found in various biologically active compounds such as sulfoxide and sulforaphane with significant Nrf2 activation [124,125,126]. Sulfinyl moiety has been linked to anticancer and chemopreventive action of sulforaphane [127,128].

#### 2.2.1. Sulfoxide Derivatives 

**Scheme 7:**
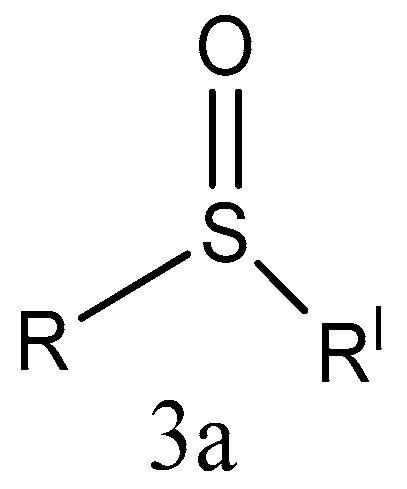
Structure of sulfoxide.

Sulfoxides are chemical compounds in which a sulfinyl (SO) group is bonded to two carbon atoms (Scheme 7) [129]. They exhibit anti-inflammatory and antioxidant activities [130,131]. Dimethyl sulfoxide and alliin are good examples of sulfoxides with Nrf2 activation ability [125,126,127,128,129].

**Scheme 8:**
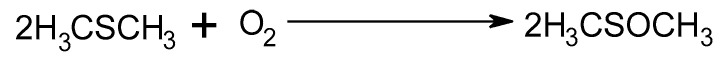
Synthesis of dimethyl sulfoxide.

Dimethyl sulfoxide (DMSO) is an organosulfur compound in which a sulfinyl group is bonded to two methyl groups [132]. DMSO is usually synthesized by the oxidation of dimethyl sulfide (Scheme 8) [133].

Due to its amphiphilic properties, comprising its high miscibility with water and its ability to dissolve lipophilic compound, DMSO is widely employed as a vehicle for drugs [125]. One essential feature of a good vehicle is being pharmacologically inert, a condition generally achieved when low DMSO concentrations are used (commonly DMSO 0.1% *v*/*v* is considered a safe concentration in cell biology). Expectedly, the effect of higher concentrations of DMSO (in the range of 1–20 % *v*/*v*) is not negligible anymore. At these concentrations DMSO has been shown to display antioxidant and anti-inflammatory effects [130,131].

Particularly, Liang and co-workers [125] reported that DMSO (**23**) (Table 1) stimulates the activation of Nrf2 and enhances its DNA binding in human umbilical vein endothelial cells (HUVEC). DMSO was found to be non-toxic to HUVEC even at very high concentrations [134]. DMSO-dependent HO-1 induction is mediated by Nrf2 pathway activation and DMSO also induces HO-1 expression and activity in a dose and time-dependent pattern in the cells. Furthermore, DMSO (**23**) has been reported to activate HO-1 protein expression via cJun-N-terminal kinase (JNKs)-dependent pathways, increase the nuclear translocation of Nrf2, and promote the antioxidant response element (ARE) binding of Nrf2 [125].

#### 2.2.2. Vinyl Sulfoxide Derivatives 

Shim et al. [135] reported that vinyl sulfoxide derivatives (compound **24** and **25**) (Table 1) exhibit moderate HO-1 inducing activities (69.8 and 62.4% respectively) as a measure of Nrf2 activation but do not inhibit NO production. Woo et al. [99] also reported that vinyl sulfoxide derivative (**26**) displays a moderate HO-1 inducing activity (133.5%) and Nrf2 activation when compared with vinyl sulfone (257.5%) and chalcone (164.4%) analogues. Shim and co-workers [135] reported that the addition of OH-, Ome-, and F- groups affects the HO-1 induction of compounds **24** and **25**, and the presence of hydroxyl and keto forms produces the same effect on HO-1 induction.

#### 2.2.3. Sulfoximine Derivatives

**Scheme 9:**
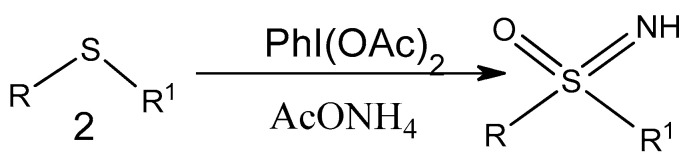
Synthesis of sulfoximine.

Sulfoximines are aza-analogues of sulfones in which the sulfinyl group is bonded to a nitrogen. They possess several biological activities, as extensively reviewed in [136,137]. Sulfoximine is synthesized from sulfides via one-pot O- and N-transfers (Scheme 9) [138].

Carlstrom et al. [100] reported that vinyl sulfoximine derivatives (compounds **27** and **28**) (Table 1) activate transcriptional factors such as Nrf2, HIF1, and NFĸB via the pTRAF platform in comparison with dimethyl fumarate. Compounds **27** and **28** activate Nrf2 and downstream transcripts in vitro with a very minimal off-target effect on HIF1 and NFĸB. In HEK293 cells, GCLM, GSTA4, and NQO1 which are known Nrf2-activated transcripts display similar regulation with both compound **28** and dimethyl fumarate. Microglia exhibits a diverse Nrf2-pattern, while oligodendrocytes show a more overlapping response to compound **28** and dimethyl fumarate. Compound **28** exhibits very high Nrf2-activation potency upon evaluation in pTRAF-transfected HEK293 cells. When compared with dimethyl fumarate, compound **28** possesses a better Nrf2-specific activating profile and the oligodendrocyte phenotype in terms of the proliferation of pre-myelinating cells is influenced by this factor. Moreover, the off-target effects of compound **28** are more limited when compared to dimethyl fumarate in HEK293 cells. Compound **28** exhibits improved Nrf2 activation when co-stimulated with tumor necrosis factor [100]. Treatment with a sulfoximine derivative L-buthionine-S-R-sulfoxime reportedly upregulated γ-glutamylcysteine ligase mRNA and L-cys/L-cys2 transporter via the activation of Nrf2 and other pathways in the striatum [139].

### 2.3. Oltipraz

Oltipraz (**29**) (Table 1) is an organosulfur compound that belongs to the class of synthetic dithiolethiones [140]. Being a chemopreventive and anti-angionic agent, it protects the body from several carcinogenic insults via the induction of detoxification enzymes [141]. It is synthesized by the reaction of methyl2-methyl-3-(pyrazine-2-yl)-3-oxopropionate with phosphorus pentasulfide in a mixed solvent of xylene and toluene [142]. Oltipraz (**29**) has been reported as an effective activator of the Nrf2 signaling pathway, being a soft electrophile, it activates Nrf2 and stimulates the production of many phase II detoxification enzymes [141]. It induces the expression of antioxidant genes such as quinone oxidoreductase (NQO1) via the activation of Nrf2 and constitutive androstane receptor (CAR) [143]. Oltipraz (**29**) also hinders fat-induced insulin resistance and obesity via the upregulation of Nrf2 in C57BL/6J mice [144] and prevents high glucose-induced apoptosis and oxidative stress via activation of the Nrf2/NQO1 pathway in RSC96 cells [145]. Atilano-Roque et al. [146] observed the effect of Nrf2 activation by oltipraz in the amelioration of injury in the human kidney cells. It was discovered that oltipraz could be a potential therapy for the enhancement of nephroprotectant effects for cisplatin-exposed kidney injury patients. Ramos-Gomez et al. [147] reported that the effects of oltipraz and Nrf2 genotype on benzo[a]pyrene-DNA adducts and tumor yield are interactive. This suggests that the anticarcinogenic activity of oltipraz in mice depends on its ability to modulate the Nrf2–regulated genes since the loss of Nrf2 in nrf2-deficient mice abrogates its chemopreventive effects. Iida et al. [148] corroborated the fact that activation of Nrf2 is pivotal to the chemopreventive potency of oltipraz (**29**) against the carcinogenesis of urinary bladder and enhancement of the detoxification of carcinogens.

**Table 1:** Synthetic organosulfur compounds and their Nrf2 activation.

Entry	Compounds	Effective Concentration/Dose	Biological Activity	Study Model	Targeted Diseases	Ref
4	**SULFONE DERIVATIVES** 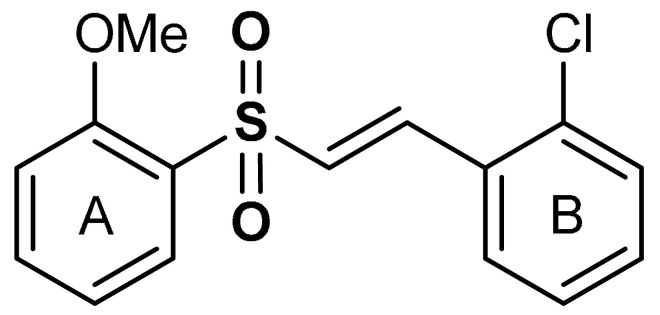 (*E*)-1-chloro-2-(2-((2-methoxyphenyl)sulfonyl)vinyl)benzene	1–5 µM	Antioxidant, anti-inflammatory	Microglia, Parkinson’s disease animal model	Parkinson’s disease	[98]
		1–10 µM	Antioxidant, neuroprotection	MPTP-induced Parkinson’s disease mouse model	Parkinson’s disease	[99]
		10 µM	Neuroprotection	HEK293 cells	Traumatic brain injury	[100]
5	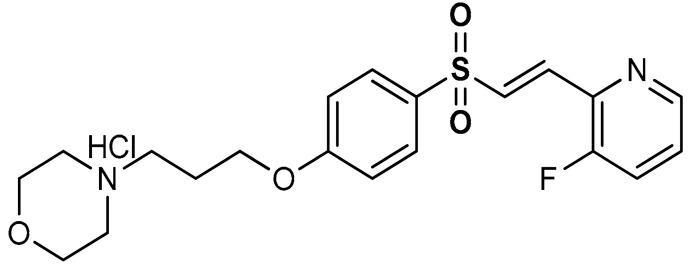 (*E*)-4-(3-(4-((2-(3-fluoropyridin-2-yl)vinyl)sulfonyl)phenoxy)propyl)morpholine hydrochloride	10 µM	Antioxidant, neuroprotection	MPTP-induced Parkinson’s disease mouse model	Parkinson’s disease	[102]
6	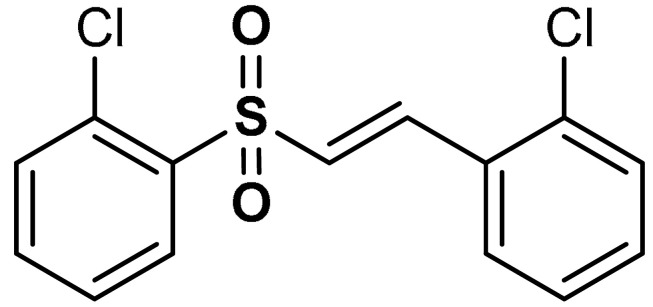 (*E*)-1-chloro-2-(2-((2-chlorophenyl)sulfonyl)vinyl)benzene	0.5–1 µM	Antioxidant, neuroprotection	PC12 Cells	Oxidative stress	[32]
7	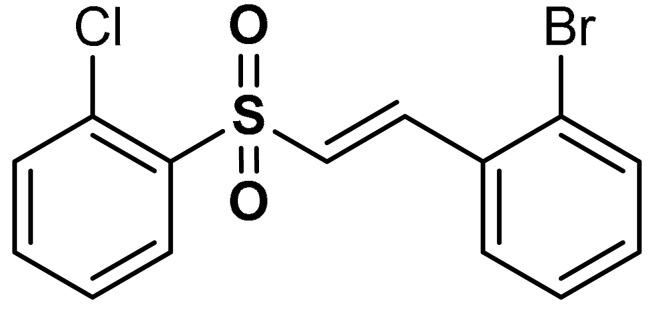 (*E*)-1-bromo-2-(2-((2-chlorophenyl)sulfonyl)vinyl)benzene	0.5–1 µM	Antioxidant, neuroprotection	PC12 Cells	Oxidative stress	[32]
8	**SULFONAMIDE DERIVATIVES** 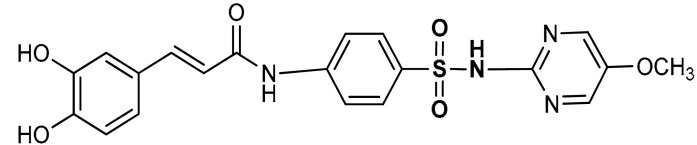 (*E*)-3-(3,4-dihydroxyphenyl)-*N*-(4-(*N*-(5-methoxypyridin-2-yl)sulfamoylphenyl)acrylamide	50–200 µM	Antioxidant	A549 Cells	Oxidative stress	[111]
9	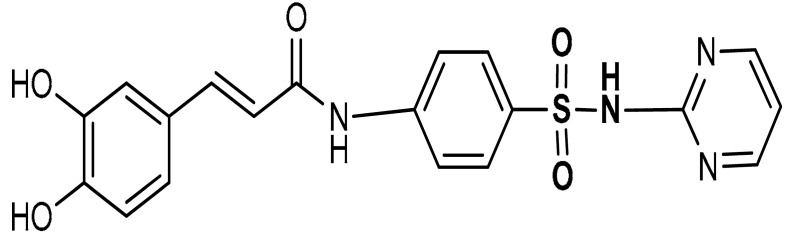 (*E*)-3-(3,4-dihydroxyphenyl)-*N*-(4-(*N*-pyrimidin-2-yl)sulfamoylphenyl)acrylamide	50–200 µM	Antioxidant	A549 Cells	Oxidative stress	[111]
10	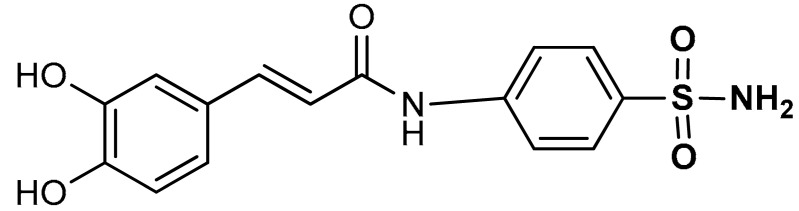 (*E*)-3-(3,4-dihydroxyphenyl)-*N*-(4-sulfamoylphenyl)acrylamide	50–200 µM	Antioxidant	A549 Cells	Oxidative stress	[111]
11	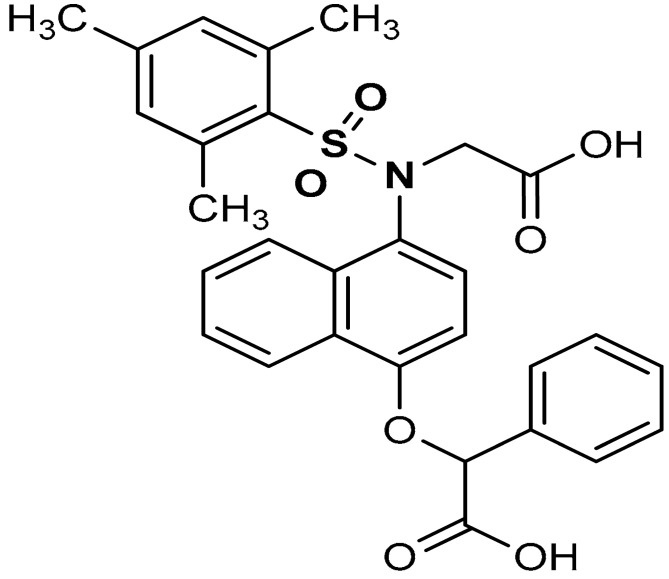 2-((4-(*N*-(carboxymethyl)-2,4,6-trimethylphenylsulfonamido)naphthalen-1-yl)oxy)-2-phenylacetic acid	0.58 µM	Antioxidant, Keap1–Nrf2 PPI inhibition	RAW264.7 Cells	Inflammation	[94]
12	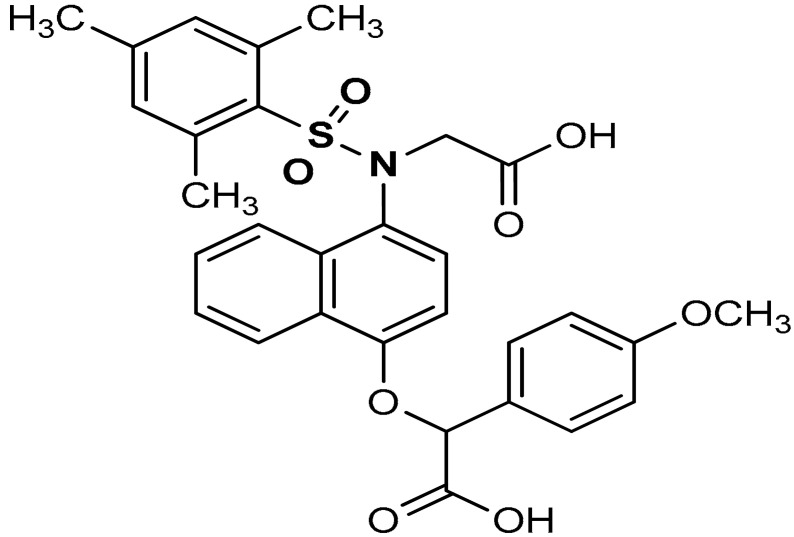 2-((4-(*N*-(carboxymethyl)-2,4,6-trimethylphenylsulfonamido)naphthalen-1-yl)oxy)-2-(4-methoxyphenyl)acetic acid	≥500 µL	Antioxidant, Keap1–Nrf2 PPI inhibition	RAW264.7 Cells. HepG2 Cells, mice, rat	Inflammation	[94]
13	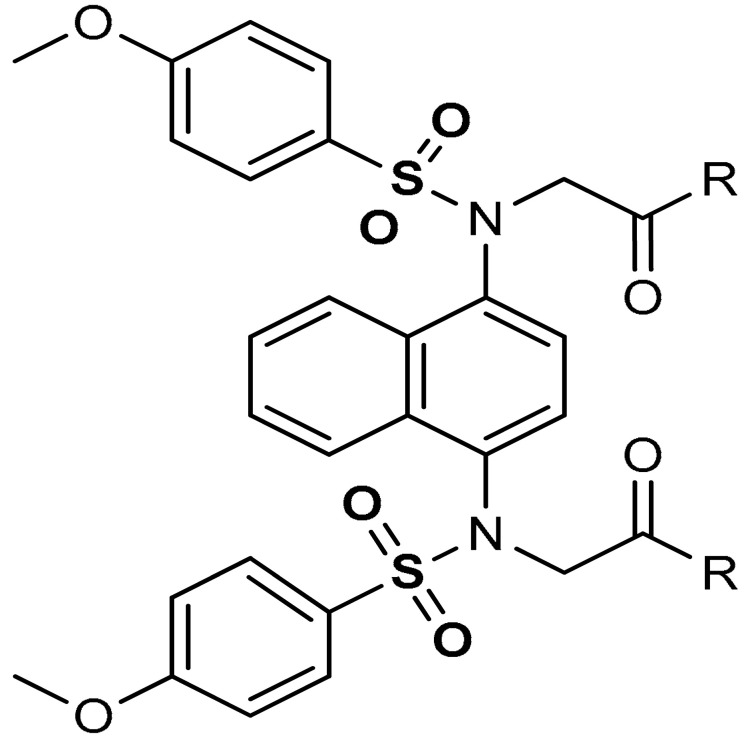 2,2′-(naphthalene-1,4-diylbis(((4-methoxyphenyl)sulfonyl)azanediyl))diacetic acid (R=OH)	≥500 µL	Antioxidant, Keap1–Nrf2 PPI inhibition	RAW264.7 Cells. HepG2 Cells, mice, rat	Inflammation	[94]
14	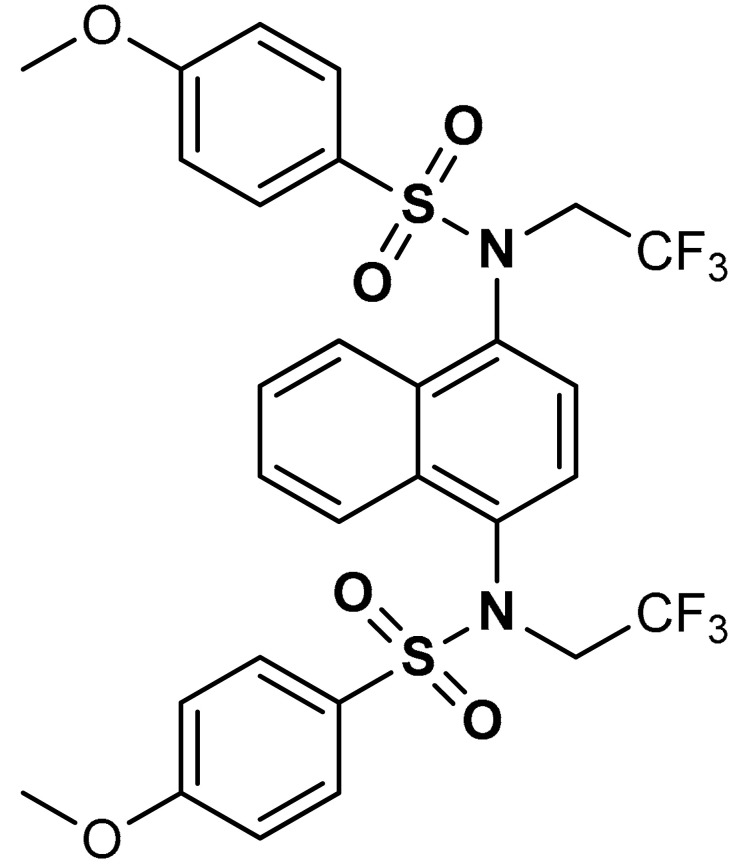 *N*-(4-((4-methoxy-*N*-(2,2,2-trifluoroethyl)phenyl)sulfonamido)isoquinolin-1-yl)-*N*-((4-methoxyphenyl)sulfonyl)glycine	≥500 µL	Antioxidant, Keap1–Nrf2 PPI inhibition	RAW264.7 Cells. HepG2 Cells, mice, rat	Inflammation,	[94]
15	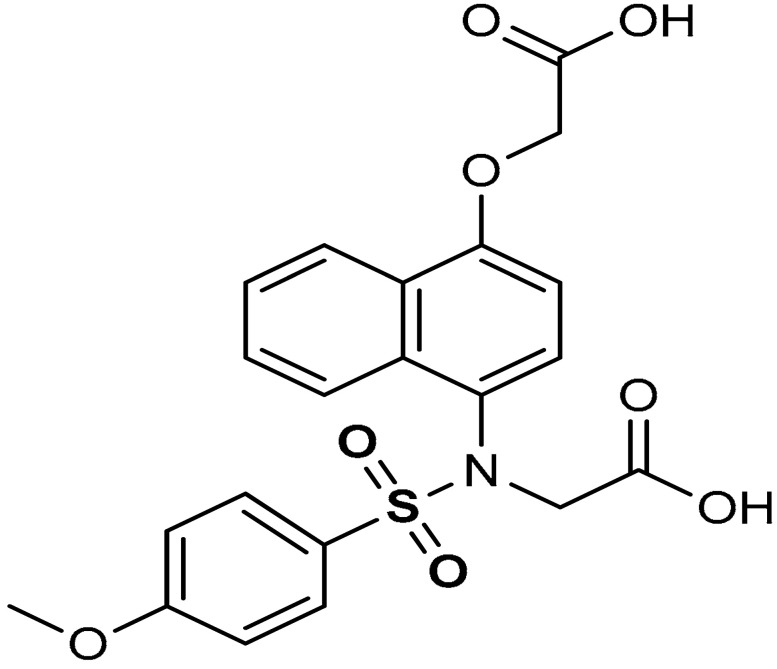 2-(*N*-(4-(carboxymethoxy)naphthalen-1-yl)-4-methoxyphenylsulfonamido)acetic acid	8.19 µM	Antioxidant, Keap1–Nrf2 PPI inhibition	RAW264.7 Cells	Inflammation	[94]
16	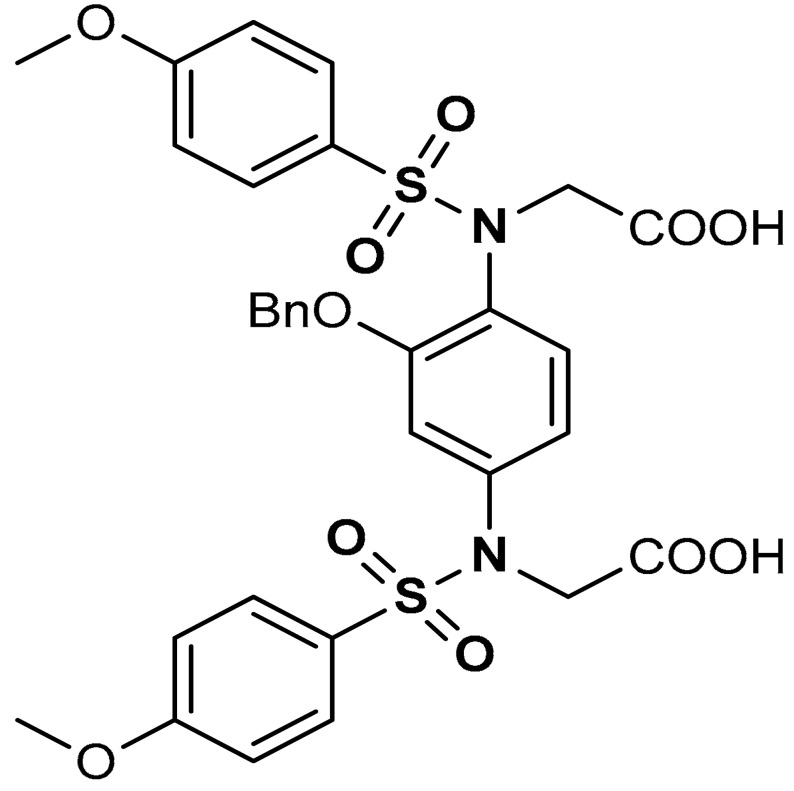 2,2′-((2-(Benzyloxy)-1,4-phenylene)bis(((4- methoxyphenyl)sulfonyl)azanediyl))diacetic acid	10–100 µM	Keap1–Nrf2 PPI inhibition, Antioxidant,	Hepa1c1c7 mouse hepatic cells	Cytotoxicity	[114]
17	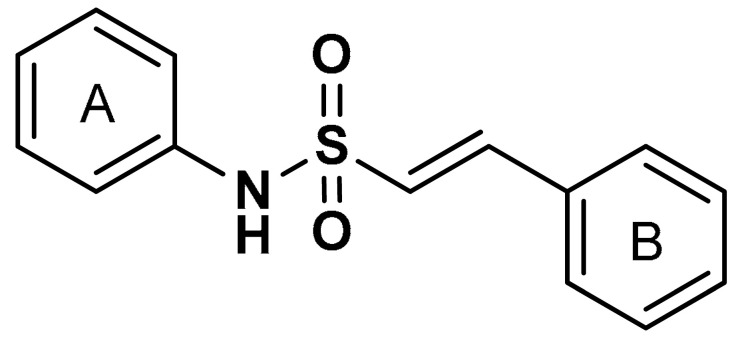 (*E*)-N,2-diphenylethenesulfonamide	>10 µM	Antioxidant, anti-inflammatory, neuroprotection	MPTP-induced PD mouse model	Parkinson’s disease	[115]
18	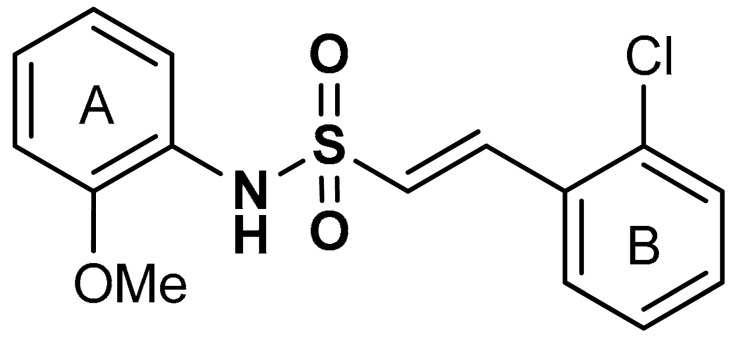 (*E*)-2-(2-chlorophenyl)-N-(2-methoxyphenyl)ethenesulfonamide	6.35 µM	Antioxidant, anti-inflammatory, neuroprotection	MPTP-induced PD mouse model	Parkinson’s disease	[115]
19	**SULFONATE DERIVATIVES** 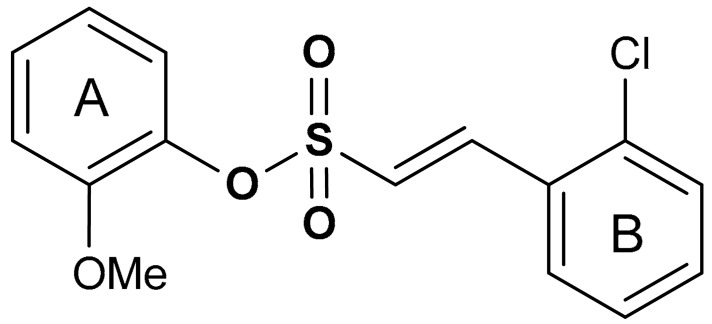 (*E*)-2-methoxyphenyl 2-(2-chlorophenyl)ethenesulfonate	0.076 µM	Antioxidant, anti-inflammatory, neuroprotection	MPTP-induced PD mouse model	Parkinson’s disease	[115]
20	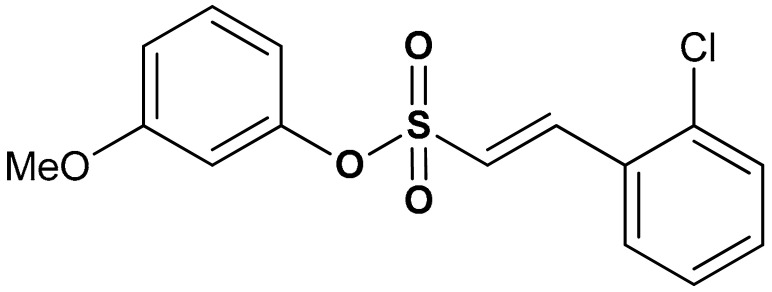 (*E*)-3-methoxyphenyl 2-(2-chlorophenyl)ethenesulfonate	0.165 µM	Antioxidant, anti-inflammatory, neuroprotection	MPTP-induced PD mouse model	Parkinson’s disease	[115]
21	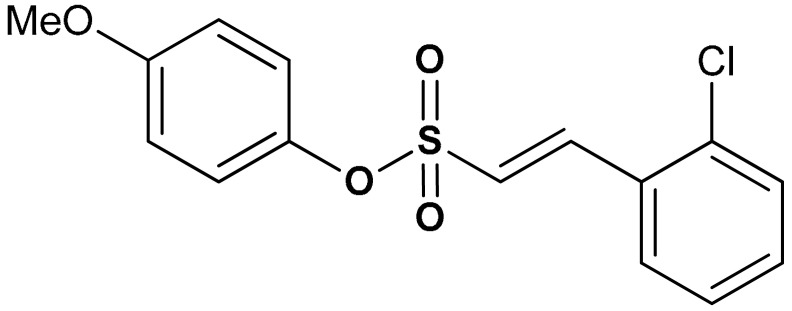 (*E*)-4-methoxyphenyl 2-(2-chlorophenyl)ethenesulfonate	0.237 µM	Antioxidant, anti-inflammatory, neuroprotection	MPTP-induced PD mouse model	Parkinson’s disease	[115]
22	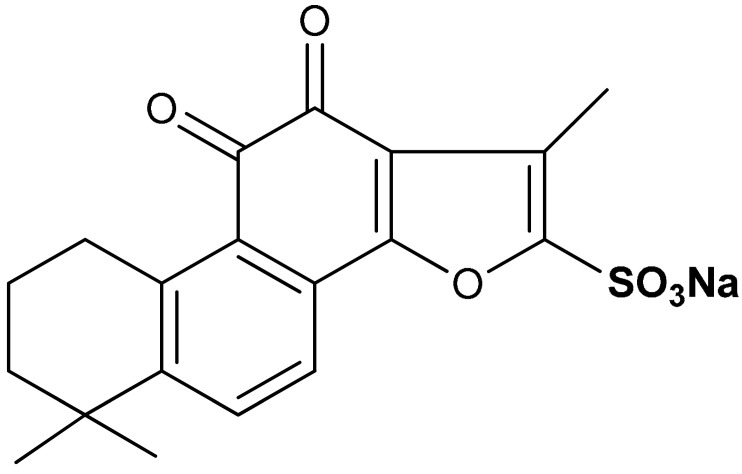 sodium 1,6,6-trimethyl-10,11-dioxo-6,7,8,9,10,11-hexahydrophenanthro [1,2-b]furan-2-sulfonate	10 µg/mL	Antioxidant	RAW 264.7 Cells, MRC-5 Cells	Silicosis	[121]
23	**SULFOXIDE DERIVATIVES** 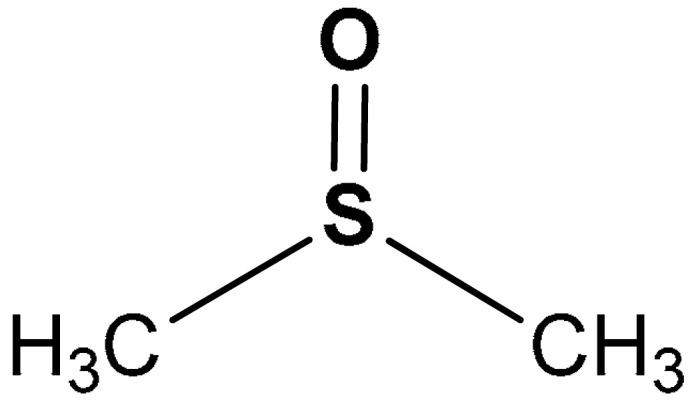 (methylsulfinyl)methane	0.1–0.8%	Antioxidant, anti-inflammatory	Human umbilical vein endothelial cells (HUVECs)	Oxidative stress	[125]
24	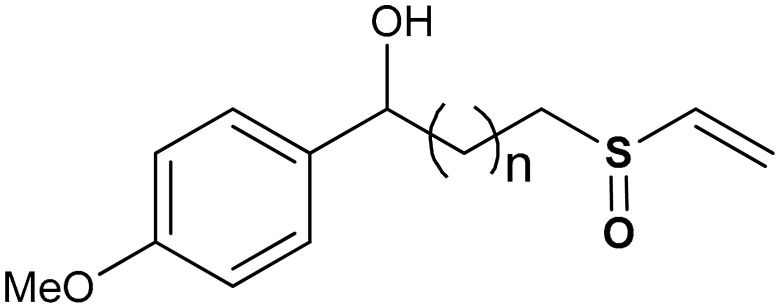 1-(4-methoxyphenyl)-3-(vinylsulfinyl)propan-1-ol	20 µM	Antioxidant, Nrf2 activation, HO-1 induction	BV-2 Cells	Parkinson’s disease	[135]
25	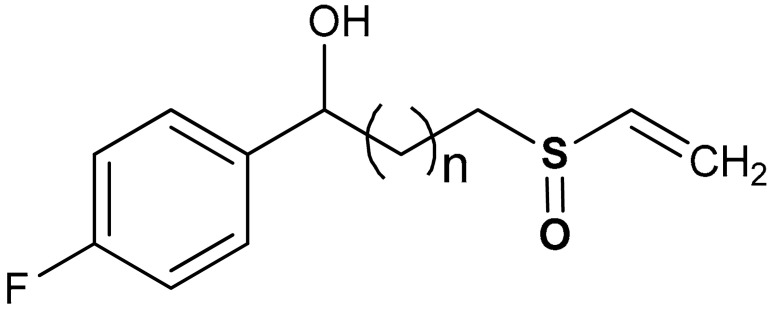 1-(4-fluorophenyl)-3-(vinylsulfinyl)propan-1-ol	20 µM	Antioxidant, Nrf2 activation, HO-1 induction	BV-2 Cells	Parkinson’s disease	[135]
26	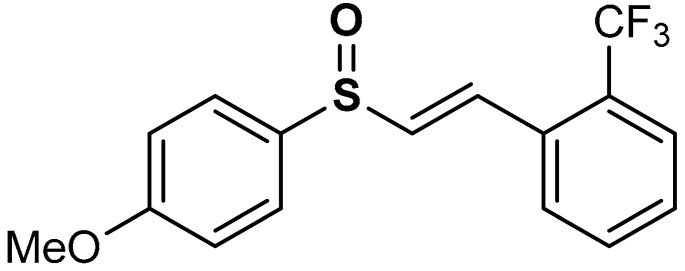 (*E*)-1-(2-((4-methoxyphenyl)sulfinyl)vinyl)-2-(trifluoromethyl)benzene	20 µM	Neuroprotection, Antioxidant	BV-2 Cells	Parkinson’s disease	[99]
27	**SULFOXIMINE DERIVATIVES** 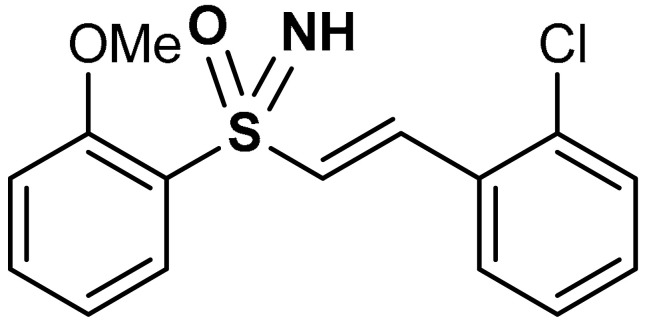 (*E*)-1-chloro-2-(2-(2-methoxyphenylsulfonimidoyl)vinyl)benzene	10 µM	Nrf2 activation, Antioxidant	HEK293 Cells	Traumatic brain injury (TBI)	[100]
28	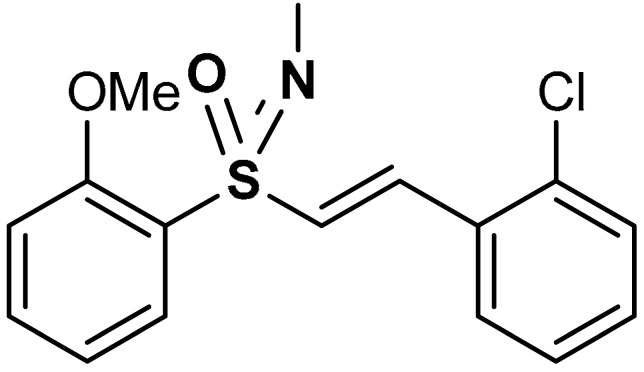 1-chloro-2-{(*E*)-2-[*S*-(2-methoxyphenyl)-*N*-methylsulfonimidoyl]ethenyl}benzene	10 µM	Nrf2 activation, Antioxidant	HEK293 Cells	Traumatic brain injury (TBI)	[100]
29	**OLTIPRAZ** 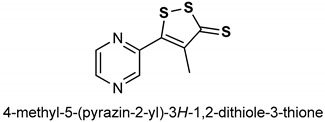	150 mg/kg	Antioxidant, Nrf2 activation	HepG2 cells and C57BL/6 mouse liver	oxidative stress	[143]
		0.75 g/kg	Antioxidant	C57BL/6J mice	Obesity, insulin resistance, Oxidative stress	[144]
		20 µM	Antioxidant	RSC96 cells	Oxidative stress, Apoptosis	[145]
		12 µM	Antioxidant. Nrf2 activation	HEK293 cells	Kidney cell injury	[146]
		500 mg/kg	Antioxidant, Chemoprevention	Mice	Cancer	[147]
		250 mg/kg	Anticancer, Antioxidant	Mouse urothelial cells	Urinary bladder carcinogenesis	[148]

## 3. Nrf2-Activating Natural Organosulfur Compounds

### 3.1. Isothiocyanates

Isothiocyanates (ITCs) are organosulfur compounds majorly obtained as hydrolysis products of glucosinolates from cruciferous vegetables. However, their synthetic derivatives can be obtained by the substitution of the oxygen atom in the isocyanate moiety with a sulfur atom. Owing to their chemical diversity and biological activities, ITCs have been widely studied in food science and medical research [149,150]. Naturally occurring ITCs such as benzyl isothiocyanate (BITC), allyl isothiocyanate (AITC), phenylethyl isothiocyanate (PEITC), and sulforaphane (SFN) activate the Nrf2/Keap1 signaling pathway. Cheng et al. [151] reported that moringa isothiocyanate (**30**) (Table 2) suppresses oxidative stress and inflammation in high glucose induced renal cells via the activation of Nrf2/ARE signaling and upregulation of Nrf2 target genes.

#### 3.1.1. Benzyl Isothiocyanate

Benzyl isothiocyanate (BITC) (**31**) (Table 2) attenuates obesity-induced hyperglycemia via the enhancement of Nrf2-mediated antioxidant IRS-1/AKT/TBC1D1 signaling in muscles [152]. EL Badawy et al. [153] reported that BITC modulates oxidative stress, inflammation and apoptosis via the activation of Nrf_2_/HO-l and NF-ĸB signaling pathways in gastric injury in rats. BITC inhibits the production of excessive superoxide in inflammatory leukocytes and also exhibits significant chemo preventive effects via the activation of the Nrf2/Keap1 signaling pathway [154,155].

#### 3.1.2. Allyl Isothiocyanate

Allyl isothiocyanate (AITC) (**32**) (Table 2) exhibits chemopreventive actions via Nrf2/Keap1 signaling and induces phase II cytoprotective and detoxifying enzymes [155]. AITC attenuates inflammation and oxidative stress via the modulation of Nrf2/HO-1 and NF-ĸB pathways, thereby ameliorating traumatic brain injury in mice [156]. Zhang et al. [157] reported that AITC elevates the expression of multidrug resistance-related protein 1 (MRP1) in cigarette smoke extract-stimulated human bronchial epithelial cells via the activation of the JNK/Nrf2 pathway. It also reduces spontaneous degradation in hepatocyte and attenuates acetaminophen-induced liver injury via the activation of Nrf2, thereby making it a potential drug for liver-related injuries [158].

#### 3.1.3. Phenylethyl Isothiocyanate

Phenylethyl Isothiocyanate (PITC) (**33**) (Table 2) is an effective activator of cytoprotective pathways such as Nrf2 and heat shock factor 1 (HSF1). However, it helps the accumulation of reactive oxygen species at high concentration resulting in cytotoxicity [159]. PITC activities Nrf2 and significantly upregulates mRNA and protein levels of the HO-1, NQO1 and γ-glutamyl cysteine synthetase (γGCS) in cultured fibroblast [160]. Nrf2 plays an essential role in the antioxidant and anti-inflammatory effect of PEITC as Nrf2 knockout reportedly attenuated its anti-inflammatory activities [161]. Moreover, the chemopreventive actions of PITC have been linked to the activation of NrF2/ARE signaling. It also activates ARE-mediated phase II drug metabolism expressions via the NrF2 and C-June N-terminal Kinase 1 (JNK1) pathways [162].

#### 3.1.4. Sulforaphane

Sulforaphane (SFN) (**34**) (Table 2) is a sulfinyl group-containing aliphatic lipophilic organosulfur compound, it is a broccoli-derived phytochemical with several biological activities, especially Nrf2 activation and chemopreventive abilities. Sulforaphane (1-isothiocyanato-(4R)-(methylsulfinyl)butane: CH_3_S(O)(CH_2_)_4_^−^N=C=S) being a phytochemical-based supplement, is by comparison a more potent Nrf2 activator than resveratrol, curcumin, and silymarin [163]. Its lipophilicity and lower molecular weight (MW = 177.29) are responsible for its higher bioavailability compared to the polyphenol-based dietary supplements with Nrf2-activating ability [163]. SFN exhibits antioxidant and anti-inflammatory activities via Nrf2/GSH system [164,165]. SFN induces Nrf2 by covalently binding to Keap1 [166,167]. Kubo et al. reported [168] that, via the induction of Nrf2/ARE/Prdx6 activity, sulforaphane reactivates cellular antioxidant defense during oxidative stress and aging. SFN in a dose-dependent fashion augments the expression of peroxiredoxin-6 (Prdx6), glutathione S-transferase (GST) and catalase (CAT) and halts their Nrf2 dysregulation [168]. It strengthens Nrf2/DNA binding and enhances promoter activities by increasing the expression and nuclear translocation of Nrf2. SFN in a dose-dependent manner also protects cells from UVB-induced toxicity, attenuates HJloss of Prdx6 due to dysregulation of Nrf2/ARE and therefore combats aging-related diseases [168]. Clarke et al. [169] reported that SFN is easily metabolized and distributed to target tissues in both wild-type and Nrf2-/-Mice. It is majorly present in tissues as N-acetylcysteine, glutathione and cysteinyl conjugates not as free molecule. Although SFN activates the Nrf2 pathway, Nrf2 is not essential for the metabolism and tissue distribution of SFN. Based on a quantitative LC-MS/MS analysis, SFN was recommended as an effective dietary chemopreventive agent for colon and prostate cancer [169]. Thimmulappa et al. [170] reported that SFN exerts its chemopreventive effect via activation of the Nrf2 pathway and consequent induction of phase II enzymes, thus providing protection against a broad spectrum of exogenous and endogenous toxicants. SFN attenuates the formation of colonic aberrant crypt foci in carcinogen-treated rats [171]. Using oligonuncleotides, Thimmulappa and co-workers [170] observed that SFN induces Nrf2-regulation of genes encoding antioxidant enzymes such as GST, UDP-glucuronosyltransferases (UGT), γ-glutamylcysteine synthetase (GCS), NQO1, genes encoding cellular NADPH regenerating enzymes, xenobiotic metabolizing enzymes, and antioxidants. Recently, Xiong and co-workers [172] reported that SFN attenuates methamphetamine (METH)-induced apoptosis and oxidative damage by exerting a neuroprotective effect via the activation of the Nrf2-mediated pathway in neuronal cells. It also significantly increases cell viability in PC12 cells post-METH exposure via the activation of the Nrf2/HO-1/GCS pathway. SFN is an essential and functional food-derived supplement for ameliorating neuronal oxidative damage due to METH-induced neurotoxicity [172]. Morimitsu et al. [173] reported that 6-methylsulfinylhexyl isothiocyanate (6-HITC) (**35**) (Table 2), a broccoli isolate and an analogue of sulforaphane, activates the Nrf2-dependent detoxification pathway. The isolate 6-HITC (**35**) activates antioxidant response element (ARE), and induces nuclear localization of Nrf2 and the gene expression of the phase II enzymes. It also induces πGSTP1 and αGSTA1 isozymes in RL34 cells and hepatic phase II detoxification enzymes in the body more potently than SFN [173].

### 3.2. Sulfur-Containing Amino Acids and Derivatives

Sulfur-containing amino acids such as methionine, taurine, homocysteine, and cysteine are abundant in plants and animals with numerous biological activities [174]. They exhibit significant antioxidant and anti-inflammatory effects [174,175]. Sulfur-containing amino acids are metabolically interconnected, therefore a clear discrimination of their actual contribution to a biological effect cannot always be achieved. For instance, homocysteine is a product of the methionine cycle. Homocysteine undergoes two possible fates: it can i) be re-methylated to methionine, thus closing the methionine cycle; or ii) enter the transsulfuration pathway and be sequentially processed by cystathionine β-synthase (CBS) and cystathionine γ-lyase (CSE), thus leading to the generation of cysteine. In turn, cysteine is the precursor for other physiologically relevant thiols, such as taurine and glutathione. Another possible fate of cysteine is to be catabolized to 3-mercaptopyruvate (3-MP) by cysteine aminotransferase. The 3-MP is then bio-converted to pyruvate by 3-mercaptopyruvate sulfur transferase (3MST), in a reaction that leads to the formation of persulfides (R-SSH) as co-products, such as glutathione persulfide (GSSH) and cysteine persulfide [176]. Interestingly, persulfides are potent electrophiles, which have been shown to exert an antioxidant effect on mouse neuroblastoma cells, by directly targeting Keap1 and thereby inducing Nrf2 translocation into the nucleus [177]. Hence, the effect of sulfur-containing amino acids on the activation of the Nrf2 pathway is, at least in part, mediated by persulfides, which through S-sulfhydration of Keap1 induce activation of Nrf2 and consequent activation of the antioxidant response.

#### 3.2.1. Methionine

L-Methionine (2-amino-4-(methylthio)butanoic acid) (**36**) (Table 2) is an essential sulfur-containing amino acid which helps in the stimulation of glutathione synthesis and depression of ROS accumulation [178,179]. It is an essential amino acid and through the trassulfuration pathway, a branch of the methionine cycle, leads to the biogeneration of cysteine and taurine. In turn, cysteine is a building block for the synthesis of GSH, the most abundant antioxidant species in the cell [180]. Given its key role in the bio-generation of endogenous sulfur species, it is not surprising that methionine has been appreciated for its antioxidant and anti-inflammatory effects [181,182]. Wang et al. [183] reported that the cytoprotective effect of methionine can be associated with the activation of the Nrf2/ARE pathway, as shown working in the Wistar rat model. Particularly, the activation of the Nrf2/ARE pathway by methionine normally occurs within 14 days of oral administration and induces endogenous antioxidant activity that depresses ROS-mediated oxidative damages [183]. An increased methionine intake affects the mRNA level and protein expression of Keap1 and increases the translocation of Nrf2 into the nucleus as well as its expression in the cytosol [183]. Feeding growing rats with methionine induces an effective regulation of the expression of hepatic enzymes involved in the metabolism of GSH via Nrf2/ARE pathway activation. Methionine stimulates the expression of ARE-dependent antioxidants such as CAT, HO-1, SOD, and NQO1 via Nrf2 activation [183]. It has been observed that increasing the oral administration of methionine enhances the activities of these ARE-dependent antioxidants, and this implies that the induction of endogenous antioxidant response depends on the quantity of methionine available. Methionine also stimulates both the synthesis of GSH and the expression of methionine sulfoxide reductase through the Nrf2/ARE pathway [183]. In a similar study aiming to elucidate the role of methionine on the antioxidant effect of rice protein (RP) when orally administrated, adult rats were fed with RP and methionine-supplemented RP. Herein, the authors corroborated that methionine activates the Nrf2/ARE pathway through which it augments the endogenous antioxidant capacity of RP [184]. Moreover, the activation of the Nrf2/ARE pathway by methionine stimulates the expression of methionine reductase and enhances glutathione synthesis which accounts for the augmentation of endogenous antioxidant activity of RP. Methionine activates Nrf2 by inhibiting Keap1 and Cul3 which results in the up-regulation of ARE-driven antioxidant genes [184]. This notwithstanding, an entire line of research supports the hypothesis that lower methionine intake reduces ROS generation and increases overall animal longevity. For instance, Liu et al. [185] recently reported that dietary restriction of methionine in lambs affects the redox system, changes the content of methionine and its metabolites in the liver and plasma, and, eventually, induces Nrf2 signaling pathway activation. This apparent discrepancy in the role of methionine on the regulation or redox balance is reconciled by the observation that reduced GSH levels, consequent to methionine restriction, induce the activation of the antioxidant response through Nrf2 as a compensatory mechanism. This mechanism of methionine restriction to induce its biological effects through decreasing GSH levels was shown in a study, in which cysteine was added to the methionine restriction diet [186].

#### 3.2.2. Taurine

Taurine (2-aminoethanesulfonic acid) (**37**) (Table 2) is a sulfur-containing amino acid commonly found in animal tissues, bile, and large intestines [187]. For industrial purposes taurine is synthesized by the ammonolysis of isethionic acid [188]. In humans, the bio-synthesis of taurine is strictly interconnected with the transsulfuration pathway (see above), which eventually leads to the production of cysteine. Among the possible metabolic fates, cysteine can undergo oxidation to its sulfinic acid by cysteine dioxygenase. The subsequent decarboxylation to hypotaurine and oxidation by hypotaurine dehydrogenase leads to taurine [189]. Similarly to other sulfur-containing compounds, taurine has also been shown to display antioxidant and anti-inflammatory activities. For instance, taurine supplementation attenuates oxidative stress and inflammation in patients with type 2 diabetes and reduces cardiovascular risk [190,191,192]. Yang and co-workers [193] demonstrated that taurine, via the activation of the Nrf2/HO-1 pathway, decreases ROS generation and attenuates ionizing radiation-induced GC-2 cell damage [193]. Agca et al. [194] reported that taurine attenuates the severity of oxidative stress through enhancement of the expression levels of Nrf2 and HO-1, thus leading to the activation of the Nrf2/HO-1 signaling pathway in diabetic rats [194].

#### 3.2.3. Homocysteine

Homocysteine (**38**) (Table 2) is a sulfur-containing amino acid and is the end-product of the methionine cycle in the liver [195,196]. The possible fates of homocysteine are: (i) re-entering in the methionine cycle, thus undergoing re-methylation to methionine; or (ii) contributing to the generation of cysteine through the transsulfuration pathway. The transsulfuration pathway plays a pivotal role in the regulation of sulfur metabolism and has a tight connection with the antioxidant response [197]. It consists in a double-step reaction in which homocysteine is enzymatically condensed with serine (or cysteine) by CBS, thus producing cystathionine. The latter, in turn, is bio-converted to cysteine by CSE. Both CBS and CSE produce hydrogen sulfide (H_2_S) as side-product, a gaseous signaling molecule (also referred as a gasotransmitter) mediating cell protection against oxidative stress through stabilization of Nrf2 via Keap1 inhibition [42,71]. Hourihan et al. [42] reported that MEF cells exposed to NaHS, an H_2_S donor, correlated with increased Nrf2 half-life and increased expression levels of Nrf2 target genes. Most interestingly, CBS and CSE (but not 3MST) are among the genes controlled by Nrf2, thus establishing a feedback loop in which the homocysteine-derived H_2_S activates Nrf2 which in turn induces the expression of H_2_S-synthesizing enzymes [42]. The induction of CSE and CBS by Nrf2 is not surprising as they regulate the transsulfuration pathway, which is the major source of the two main physiological antioxidants, cysteine and GSH, along with H_2_S which activates antioxidant stress response via the sulfhydration of target proteins [198]. CBS and CSE enzymatic activity have also been associated with the production of reactive sulfur species, such as persufides (R-SSH) [199]. Differently from their thiol cognate which can be only nucleophilic, persulfide can be either nuclophilic or electrophilic, according to the protonation state. Persulfides owe their reactivity to the presence of an electron lone pair on the atom adjacent to the nucleophilic atom (α-effect) [200]. Consistently, persulfides have been shown to be much stronger antioxidants as compared to their corresponding thiols, by direct scavenging ROS and by activating antioxidant response, similarly to the Nrf2 pathway [201]. Persulfidation of Keap1 has been shown to promote the dissociation of Nrf2 from the Nrf2–Keap1 complex, thus inducing the expression of the downstream Nrf2-targeted genes [71,202,203]. Regardless the actual mediator of the effect of homocysteine, its role in cellular redox homeostasis and regulation of inflammation is controversial. For instance, in a study investigating the effect of chronic administration of homocysteine in a hypercholesterolemic rat model, it was shown that homocysteine treatment prevented cholesterol-induced inflammation and partly counteracted the cortical blood–brain barrier disruptions [204]. On the other hand, chronic hyperhomocysteinemia has been shown to induce long-term memory deficits in the rat model [205]. Indeed, as shown in HepG2 cells, homocysteine treatment induces oxidative stress which is responsible for the activation of the Nrf2 pathway, with consequent increased levels of ARE-associated proteins, such as HO-1 and GCL [206,207,208]. Navneet et al. [209] reported that excess of homocysteine (50 µM–10 mM) activates the Nrf2 pathway in retinal Muller glial cells, increases Nrf2 expression, and decreases oxidative stress and ROS levels [209].

#### 3.2.4. N-Acetylcysteine

N-acetylcysteine (NAC) (**39**) (Table 2) is a stable and supplement form of cysteine used for the treatment of paracetamol overdoses [210]. It possesses significant antioxidant and anti-inflammatory properties [211,212]. Jannatifar et al., in a clinical trial study, reported that oral supplementation of NAC enhances the quality of semen in asthenoteratozoospermia men by improving the expression level of Nrf2 genes and antioxidant enzymes [213]. NAC also decreases the production of malondialdehyde (MDA) and protects the sperm cells against oxidative stress via Nrf2 activation [213]. Zhou and co-workers reported that *N*-acetylcysteine amide (NACA) (**40**) (Table 2) attenuates brain injury, and increases the nuclear import of Nrf2 and the expression of Nrf2 downstream factors in mRNA and protein levels of mice at one day after traumatic brain injury [214]. NACA is antiapoptotic and attenuates oxidative stress and neuronal damage [214]. It is not clear whether NAC-mediated activation of the Nrf2 pathway is due to direct interaction with one of the redox-sensitive cysteines of Keap1. Most probably, given the poor electrophilicity of this compound, its effect is mediated by other molecular mediators. Indeed, Ezerina et al. have shown that NAC stimulates the production of persulfides, which were proposed as the actual mediators of the immediate antioxidants and cytoprotective effect of NAC [215]. Consistently, NAC has been reported to boost 3-MST activity both in isolated enzyme and SW480 cell models, increasing the production of H_2_S, likely through the formation of NAC-persulfide intermediate [216].

#### 3.2.5. Carbocysteine

Carbocysteine (^®^-2-amino-3-(carboxymethylsulfanyl))propanoic acid) (**41**) (Table 2) is a mucolytic agent with significant antioxidant and anti-inflammatory activities [217]. Pace et al. reported that carbocysteine reduces the production of ROS, increases HO-1, GSH, Nrf2, and HDAC-2 nuclear expression and activity in cigarette smoke extract-stimulated cells [218].

#### 3.2.6. Ergothioneine

Ergothioneine (**42**) (Table 2) is a sulfur-containing amino acid with potent antioxidant activities. It is a metabolite of histidine commonly found in high concentration in mushrooms amongst other foods [219]. They are produced by biosynthesis in certain species of fungi and bacteria [220]. Salama et al. [221] reported that Ergothioneine (**42**) ameliorates cisplatin-induced nephrotoxicity via the activation of Nrf2, p53 and NF-ĸB signaling and upregulation of antioxidant genes such as NQO1 and HO-1 in rats. Hseu and co-workers [222] reported that ergothioneine (**42**) attenuates UVB-induced skin damage even at nanomolar concentration by exhibiting dermato-protective activity via the induction of Nrf2/ARE-mediated antioxidant genes. In a similar development, ergothioneine reportedly alleviated inflammation, ROS production, apoptosis and senescence of fibroblast due to UVB-induced keratinocytes damage through the activation of the Nrf2/HO-1 signaling pathway [223].

### 3.3. Allicin

Allicin (**43**) (Table 2) is an organosulfur compound obtained from garlic bulbs, its taste and odor are typical of freshly crushed garlic [224]. Allicin (diallyl thiosulfinate) inhibits lipids peroxidation and exhibits antioxidant, radical scavenging, chemopreventive, and anti-inflammatory activities [225,226,227]. Bat-Chen et al. reported that allicin induces the apoptotic death of HCT-116 a colon cancer cell line via Nrf2 activation [228]. Allicin stimulates the release of cyt C, increases Bax-protein levels and decreases Bcl-2 level. It also stimulates the accumulation, translocation, and transactivation of Nrf2 to the nucleus. Interestingly, Nrf2 siRNA-transfected HCT-116 cells resist to allicin-induced inhibition of proliferation [228]. Zhang and co-workers reported that allicin exerts antioxidative and anti-inflammatory effects that prevent lipopolysaccharide (LPS)-associated injury in human umbilical vein endothelial cells (HUVEC) by regulating the endogeneous antioxidant system and inflammation via activation of Nrf2 and suppression of mitochondrial dysfunction [229]. Moreover, allicin hinders LPS-decreased cell viability, LPS-induced apoptosis, and adhesion of neutrophils to LPS-exposed HUVECs. It reduces LPS-induced oxidative stress and NF-ĸB binding activity in HUVECs. Allicin increases LPS-suppressed Nrf2 activity and regulates the expression of mitochondrial proteins and anti-inflammatory response. It modulates Nrf2 activation and increases the expression of LXRα in a dose-dependent manner [229].

**Table 2:** Natural organosulfur compounds and their Nrf2 activation.

Entry	Compounds	Effective Concentration/Dose	Biological Activity	Study Model	Targeted Diseases	Ref
30	**ISOTHIOCYANATES** 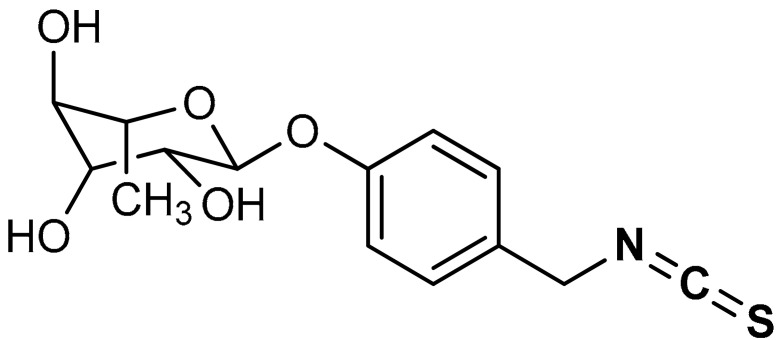 (2*S*,3*R*,4*R*,5*R*,6*S*)-2-(4-(isothiocyanatomethyl)phenoxy)-6-methyltetrahydro-2*H*-pyran-3,4,5-triol	0.4–100 µM	Antioxidant, anti-inflammatory	HepG2-C8 cells	Diabetic nephropathy, oxidative stress	[151]
31	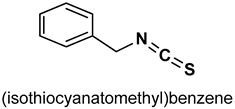	5 µM	Antioxidant	Mice	Hyperglycemia, oxidative stress	[152]
		0.7 and 1.5 mg/kg	Antioxidant, anti-inflammatory	Rat	Gastric injury	[153]
32	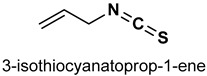	10 mg/kg	Antioxidant, anti-inflammatory	C57BL/6J mice	Traumatic brain injury	[156]
		40 µM	Antioxidant	16HBE14o-cells	COPD	[157]
		25 or 50 mg/kg	Hepatotoxicity, antioxidant	HepG2 and AML12 cells	Acetaminophen-induced liver injury	[158]
33	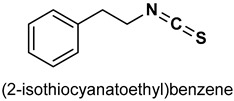	1–100 µM	Antioxiodant, cytoprotection	NIH3T3 cells	cytotoxicity	[160]
		5 and 10 µM	Antioxidant, anti-inflammatory	C57BL/6J mouse strain	Inflammation	[161]
		20 and 50 µM	Chemoprevention, antioxidant	HeLa cells	cancer	[162]
34	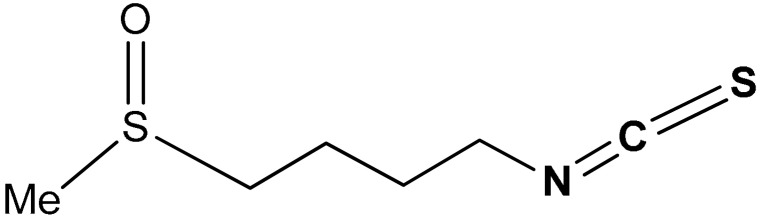 1-isothiocyanato-4-(methylsulfinyl)butane	3–8 µM	Antioxidant	Human or rat epithelial cells	Oxidative stress	[168]
		110–440 µmol/kg	Anticancer, chemoprevention	Wild-type mice	Cancer	[169]
		9 µmol/day	Anticancer, chemoprevention	Mice	Cancer	[170]
		1–10 µM	Antioxidant, neuroprotection	PC12 Cells	Oxidative stress, apoptosis	[172]
35	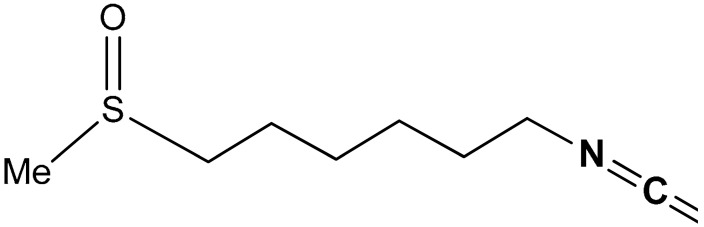 6-Methylsulfinylhexyl isothiocyanate	5 µM	Anticancer, chemoprevention	RL34 Cells	Cancer	[173]
36	**SULFUR-CONTAINING AMINO ACIDS AND DERIVATIVES** 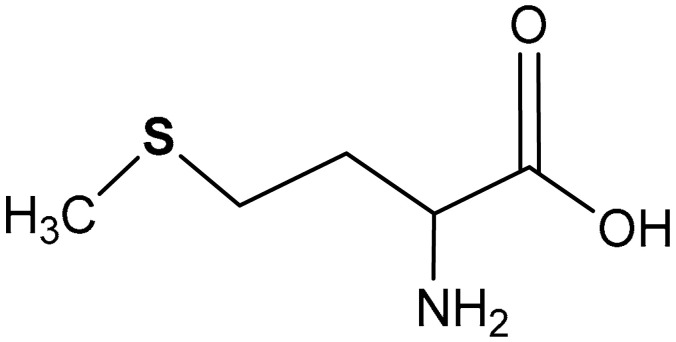 2-amino-4-(methylthio)butanoic acid	21.50–43.00 mg, 100 g^−1^ body wt	Antioxidant	Growing rats	Oxidative stress	[183]
		21.2 mg/g	Antioxidant	Growing and adult rats	Oxidative stress	[184]
		0.4–0.91%	Hepatic antioxidant	Lambs	Oxidative stress	[185]
37	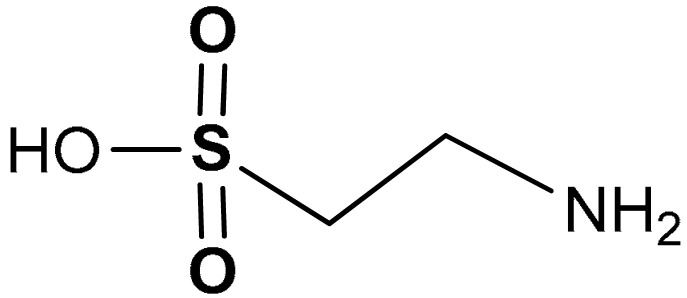 2-aminoethanesulfonic acid	10–80 mM	Antioxidant	Mouse spermatocytes (GC-2 Cells)	Oxidative stress	[193]
		2% *w*/*v*	Antioxidant	Diabetic rats	Diabetic neuropathy	[194]
38	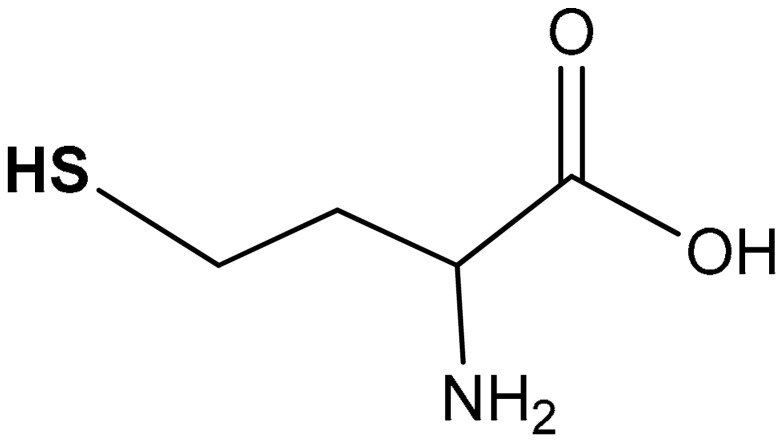 2-amino-4-mercaptobutanoic acid	50 µMD/L	Antioxidant	Hepatoma cell line (HepG2 Cells)	Oxidative stress	[206]
		50 µM	Antioxidant	Hepatoma cell line (HepG2 Cells)	Oxidative stress	[207]
		0–100 µM	Antioxidant	Hepatoma cell line (HepG2 Cells)	Oxidative stress	[208]
		50 µM–1 mM	Antioxidant	Muller glial cells	Oxidative stress	[209]
39	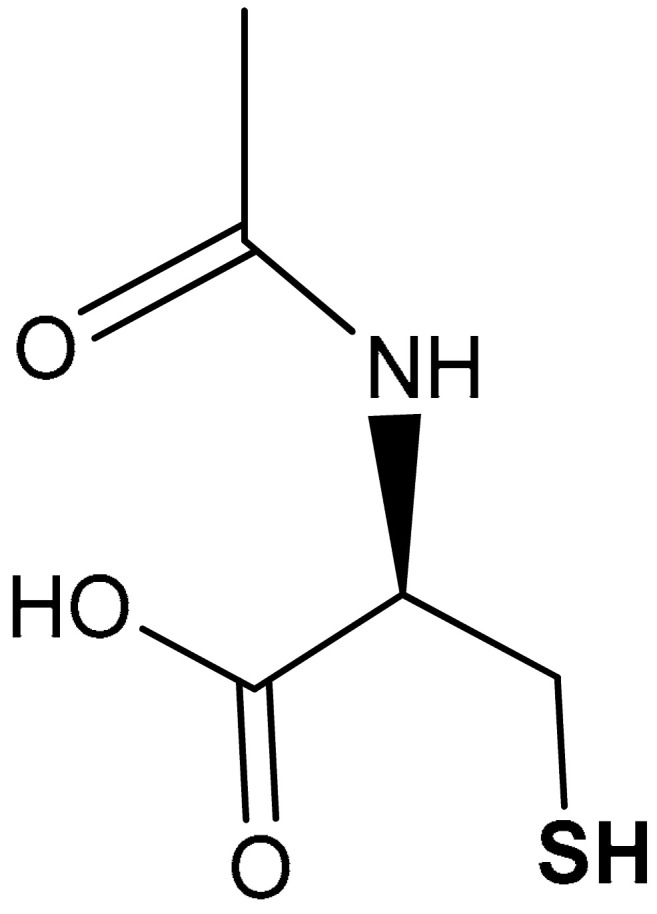 (*R*)-2-acetamido-3-mercaptopropanoic acid	600 mg	Antioxidant	Infertile men with asthenoteratozoospermia	Oxidative stress	[213]
40	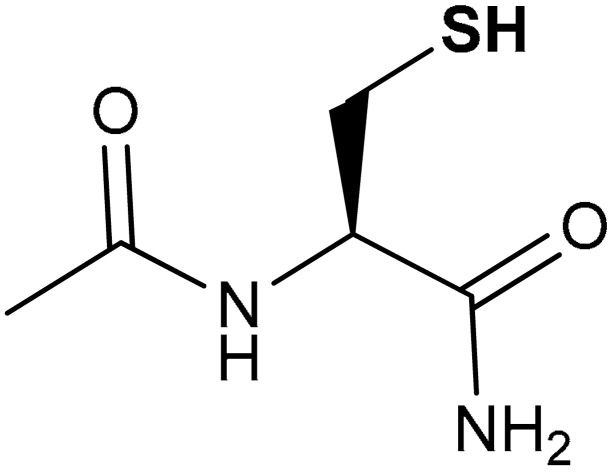 (*R*)-2-acetamido-3-mercaptopropanamide	100 mg/kg	Antioxidant, neuroprotection	Mouse model of TBI	Oxidative stress, TBI	[214]
41	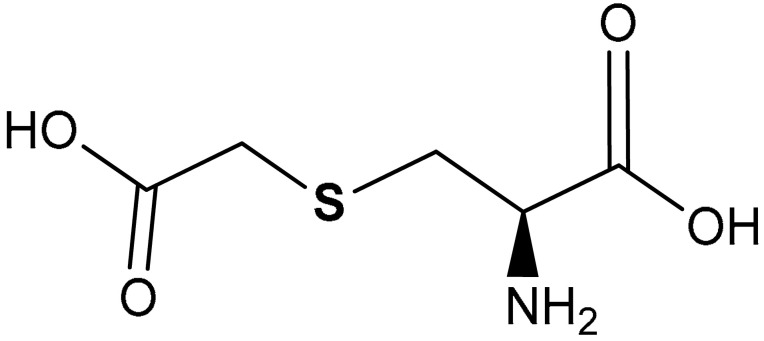 (*R*)-2-amino-3-((carboxymethyl)thio)propanoic acid	10^−4^ M	Antioxidant, Cytoprotection	Bronchial epithelial cells (16-HBE)	Chronic obstructive pulmonary disease (COPD)	[218]
42	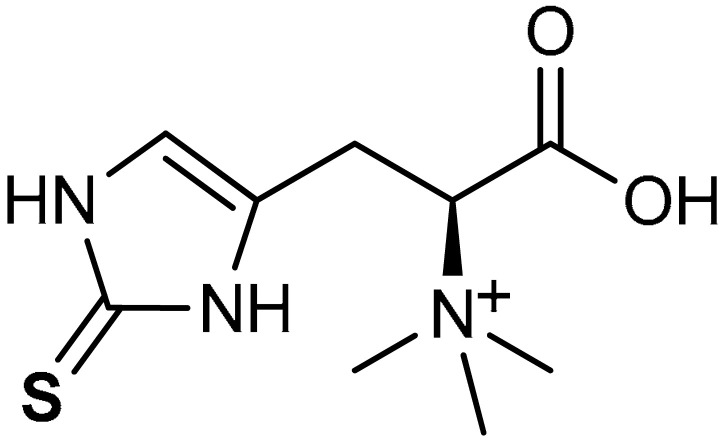 (2*S*)-3-(2-Sulfanylidene-2,3-dihydro-1H-imidazol-4-yl)-2-(trimethylazaniumyl)propanoate	70 mg/kg	Antioxidant, Anti-inflammatory	Rat	Nephrotoxicity	[221]
		125–500b nM	Antioxidant, Dermato-protection	Human keratinocytes	Skin damage, Oxidative stress	[222]
		0.1–10 mM	Antioxidant, Anti-inflammatory	Human keratinocytes	Skin damage, Oxidative stress, Inflammation	[223]
43	**ALLICIN** 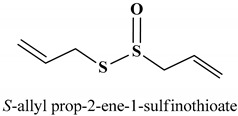	10 µg/mL	Apoptosis	Colon cancer cells (HCT-116)	Colon cancer	[228]
		40 µg/mL	Antioxidant, Anti-inflammatory	HUVECs	Oxidative stress, Inflammation	[229]

## 4. Conclusions

Nrf2 maintains redox homeostasis, regulates phase II antioxidant response, controls neuroinflammation, and remains a fascinating therapeutic target for several chronic diseases. Despite extensive research on the discovery of Nrf2 activators, only a few are approved for oxidative stress and inflammation-mediated diseases. A large body of scientific evidence shows that several organosulfur compounds are potent Nrf2 activators essential for antioxidative and anti-inflammatory purposes. This review has therefore comprehensively explored the Nrf2-activating potentials of organosulfur compounds, with particular focus on their antioxidant and anti-inflammatory effect. The ability to react with sulfhydryl (SH) groups is common amongst Nrf2 activators, and thus connects Nrf2 activation to sulfur-based chemistry, thereby creating opportunities for the discovery of novel Nrf2-activating organosulfur compounds. This review will therefore stimulate innovative research in this crucial area of medicinal chemistry.

## Abbreviations

αGSTA1	Glutathione S-transferase A1
ADME/tox	Absorption, Distribution, Metabolism, and Elimination/Toxicity
ARE	Antioxidant response element
BAX	BCL2 Associated X, Apoptosis Regulator
BBB	Blood-brain barrier
Bcl-2	B-cell lymphoma 2
BTB domain	Broad complex, tramtrack and bric-à-brac domain
β-TrCP	β-transducin repeat-containing protein
CAT	Catalase
CBP	CREB-binding protein
CBS	Cystathionine β-synthase
CDDO-Im	1-[2-cyano-3,12-dioxooleana-1,9(11)-dien-28-oyl] imidazole
CH3	Cysteine/histidine-rich domain 3
JNKs	c-Jun N-terminal kinases
COX-	2-Cyclooxygenase-2
CREB	cAMP Responsive Element Binding protein
CSE	Cystathionine γ-lyase
Cul3	Cullin3 ligase
CYP	Cytochrome P450
Cyt c	Cytochrome c
15d-	PGJ2-15-deoxy-Δ12,14-prostaglandin J2
DA	Dopaminergic
DMF	Dimethyl fumarate
DMSO	Dimethyl sulfoxide
EC_50_	Half maximal effective concentration
GCL	Glutamate-cysteine ligase
GCLM	Glutamate-cysteine ligase regulatory subunit
GCS	Gamma-glutamylcysteine synthetase
GSH	Glutathione
GSSH	Glutathione persulfide
GST	Glutathione S-transferase
GSTA4	Glutathione S-transferase Alpha 4
πGSTP1	Glutathione S-transferase π
HDAC-2	Histone Deacetylase 2
HEK293	Human embryonic kidney 293 cells
6-HITC	6-methylsulfinylhexyl isothiocyanate
HIF1	Hypoxia-inducible factor 1
H_2_O_2_	Hydrogen peroxide
H_2_S	Hydrogen sulfide
HO-1	Heme oxygenase-1
HUVEC	Human umbilical vein endothelial cells
iNOS	Inducible nitric oxide synthase
IVR	Intervening region
K_D_	Dissociation constant
Keap1	Kelch-like ECH associated protein 1
KIX	Kinase-inducible domain interacting
LC-MS/MS	High performance liquid chromatography-mass spectrometry
LPS	Lipopolysaccharides
LXRα	Liver X receptor
MAD	Malondialdehyde
3-MP	3-mercaptopyruvate
3MST	3-mercaptopyruvate sulfur transferase
Maf	Musculoaponeurotic fibrosarcoma
METH	Methamphetamine
MPTP	1-methyl-4-phenyl-1,2,3,6-tetrahydropyridine
MW	Molecular weight
NAC	N-acetylcysteine
NACA	N-acetylcysteine amide
NADPH	Nicotinamide adenine dinucleotide phosphate
NaHS	Sodium hydrosulfide
NF-κB	Nuclear factor kappa-light-chain-enhancer of activated B cells
NO	Nitric oxide
NQOI	NADPH dehydrogenase 1
Nrf2	Nuclear factor erythroid 2-related factor 2
PD	Parkinson’s disease
PPI	Protein–protein interaction
Prdx6	Peroxiredoxin 6
pTRAF	Plasmid for transcription factor reporter activation based upon fluorescence
ROS	Reactive oxygen species
RP	Rice protein
SFN	Sulforaphane
SH	Sulfhydryl group
siRNA	Small interfering RNA
SO	Sulfinyl group
SOD	Superoxide dismutase
Trx	Thioredoxin
TrxR	Thioredoxin reductase
UGT	UDP-glucuronosyltransferase
UVB	Ultraviolet B radiation
